# Improved tomato leaf disease classification through adaptive ensemble models with exponential moving average fusion and enhanced weighted gradient optimization

**DOI:** 10.3389/fpls.2024.1382416

**Published:** 2024-05-17

**Authors:** Pandiyaraju V., A. M. Senthil Kumar, Joe I. R. Praveen, Shravan Venkatraman, S. Pavan Kumar, S. A. Aravintakshan, A. Abeshek, A. Kannan

**Affiliations:** ^1^ School of Computer Science and Engineering, Vellore Institute of Technology, Chennai, India; ^2^ School of Computer Science and Engineering, Vellore Institute of Technology, Vellore, India

**Keywords:** deep learning, machine learning, image processing, ensemble learning, classification

## Abstract

Tomato is one of the most popular and most important food crops consumed globally. The quality and quantity of yield by tomato plants are affected by the impact made by various kinds of diseases. Therefore, it is essential to identify these diseases early so that it is possible to reduce the occurrences and effect of the diseases on tomato plants to improve the overall crop yield and to support the farmers. In the past, many research works have been carried out by applying the machine learning techniques to segment and classify the tomato leaf images. However, the existing machine learning-based classifiers are not able to detect the new types of diseases more accurately. On the other hand, deep learning-based classifiers with the support of swarm intelligence-based optimization techniques are able to enhance the classification accuracy, leading to the more effective and accurate detection of leaf diseases. This research paper proposes a new method for the accurate classification of tomato leaf diseases by harnessing the power of an ensemble model in a sample dataset of tomato plants, containing images pertaining to nine different types of leaf diseases. This research introduces an ensemble model with an exponential moving average function with temporal constraints and an enhanced weighted gradient optimizer that is integrated into fine-tuned Visual Geometry Group-16 (VGG-16) and Neural Architecture Search Network (NASNet) mobile training methods for providing improved learning and classification accuracy. The dataset used for the research consists of 10,000 tomato leaf images categorized into nine classes for training and validating the model and an additional 1,000 images reserved for testing the model. The results have been analyzed thoroughly and benchmarked with existing performance metrics, thus proving that the proposed approach gives better performance in terms of accuracy, loss, precision, recall, receiver operating characteristic curve, and F1-score with values of 98.7%, 4%, 97.9%, 98.6%, 99.97%, and 98.7%, respectively.

## 1 Introduction

In the dynamic landscape of modern agriculture, where crop health plays a pivotal role in global food production, the precise and timely management of plant diseases is an ongoing challenge. Among these agricultural adversaries, leaf diseases emerge as intricate and multifaceted adversaries with distinct morphological manifestations. The science of leaf disease classification, a subdomain of plant pathology, is at the forefront of efforts to combat these detrimental afflictions. This research aspires to contribute to the field of leaf disease classification through the incorporation of pioneering technologies, namely, artificial intelligence (AI) and machine learning. The criticality of early detection and accurate classification in disease management cannot be overstated. Therefore, this study seeks to harness the potential of advanced algorithms, including convolutional neural networks (CNNs) and optimization into deep learning methodologies, to revolutionize the existing approaches to leaf disease diagnosis. At its core, this research addresses the challenges posed by leaf diseases by developing a novel classification system. By utilizing image recognition and deep learning techniques, this system aims to empower agriculture practitioners and plant pathologists with a sophisticated tool for disease identification. The impact of this system extends to many applications including crop health, reaching into the realms of global food security, sustainable agricultural practices, and environmental conservation.

Deep learning is an extension to the machine learning methods such as neural networks in AI that trains the computer system to recognize the patterns similar to the human brain. Deep learning models are trained to recognize even complex patterns found in images, text, videos, and voice data to perform accurate classifications and predictions. Deep learning algorithms perform both feature extraction and feature selection automatically without needing human effort as required in machine learning algorithms for training the software based on the algorithms. A CNN is one of the most important and fundamental deep learning neural network-based algorithms used for image recognition as it provides promising and accurate results in computer vision tasks. It has many architectural implementations including LeNet, AlexNet, Visual Geometry Group (VGG), GoogLeNet, and ResNet.

Time and space are important parameters to be considered for prediction-oriented decision-making systems. The temporal and spatial data on the disease growth in tomato leaves need time series analysis on image data with temporal reasoning. Moreover, prediction using time series analysis must focus on the direction of sequence that can be performed more effectively using machine learning-based classifiers. Moving average methods support to smoothen the time series analysis by identifying the temporal data patterns more effectively. Moreover, smoothing or filtering helps to eliminate the random variations that occur in the plotted time series data. An exponential (weighted) moving average method that applies a simple recursive procedure under the hood provides flexibility to the algorithm.

Despite the presence of many works on tomato plant leaf disease detection that are found in the literature, most of the existing systems use a machine learning approach for classification without any optimizer and temporal analysis. Therefore, it is necessary to employ manual preprocessing or to apply additional machine learning-based classification algorithms or clustering algorithms when performing effective feature extraction and feature selection. Moreover, the existing systems that use time series data are not designed to give higher importance to the most recent data and also do not focus on temporal reasoning by applying temporal constraints. Moreover, the convergence of the existing deep learning algorithm employed in the detection of tomato leaf diseases is not supported by an optimization algorithm. Finally, ensemble-based classification algorithms are not employed in the classification process to enhance the detection accuracy. Therefore, it is necessary to propose a new ensemble classifier with an optimization component and a temporal data analysis component.

In this paper, an ensemble model is proposed with an exponential moving average (EMA) function with temporal constraints based on interval analysis and an enhanced weighted gradient optimizer (EWGO) in which the gradient optimizer is enhanced with temporal rules and that is integrated into VGG-16 and Neural Architecture Search Network (NASNet) CNN architectures. VGG-16 is a fine-tuned model with a 16-layer depth developed by the VGG that consists of 13 convolution and max pooling layers with three fully connected layers, and it applies stride 2. The learning rate is fixed here as 0.1. The regression-based and binary classification-based loss functions are used in this work to reduce the errors. Moreover, the NASNet mobile training methods are integrated in this ensemble model for identifying the diseases in tomato leaves by providing improved learning and classification accuracy.

NASNet is also a CNN model that consists of two types of cells, namely, the normal and the reduction cells. The EMA method is used in this ensemble model since it gives more weightage to the current data in the temporally oriented time series data. Moreover, the Plant Village dataset is used in this work to carry out the experiments for testing the ensemble model proposed in this paper. Moreover, the Plant Village dataset is a publicly available dataset consisting of 54,305 images from which 1,000 images related to tomato leaves have been extracted and used in this work for training and testing the system. The main advantages of the proposed ensemble model are the increase in classification accuracy and the reduction in error rate in the detection of tomato leaf diseases.

The main motivation for this research work is that the profession of agriculture is one of the most vital in every world economy. It is the main source of resources in our country. Nowadays, leaf disease has a great impact on the productivity of vegetables. If we cannot control the disease, then it can greatly affect the harvest. These problems provide great motivation in finding out the origin of the disease at an earlier stage to help the tomato plants grow healthily and increase their yield. Another motivation for this research is that it addresses the challenges posed by leaf diseases by developing a novel classification system. By utilizing image recognition and deep learning techniques, this system aims to empower agriculture practitioners and plant pathologists with a sophisticated tool for disease identification. The impact of this system extends beyond crop health, reaching into the realms of global food security, sustainable agricultural practices, and environmental conservation.

In this work, the Plant Village dataset is used to carry out the experiments for testing the model proposed in this paper. Moreover, the Plant Village dataset is a publicly available dataset consisting of 54,305 images from which 1,000 images related to tomato leaves have been extracted and used in this work for training and testing the system. The Plant Village dataset provides data to detect 39 different plant diseases. Moreover, the dataset contains 61,486 images of plant leaves with backgrounds. The dataset was designed using six different augmentation techniques in order to create more diverse datasets with different background conditions. The augmentations that have been used in this process include scaling, rotation, injection of noise, gamma correction, image flipping, and principal component analysis to perform color augmentation.

The main contributions of this paper are as follows:

- Proposal of an ensemble model using VGG-16 and NASNet mobile training deep learning models with an EMA function.
- Effective time series analysis using the CNN-based deep learning classifier along with an EWGO.
- Use of the Plant Village dataset for validation.
- Evaluation using suitable metrics.

The research unfolds in the following sequence: Section 2 provides a comprehensive exploration of the taxonomy and intricacies of leaf diseases. Section 3 is a detailed methodology section highlighting the technical aspects of image processing and machine learning, and the revelation of a state-of-the-art deep learning classification system designed to improve the accuracy and efficiency of leaf disease identification. In section 4, performance assessment of the proposed approach and results are compared with existing techniques. We conclude the research paper in section 5.

The VGG-16 architecture is a deep CNN designed for image classification tasks. It was introduced by the VGG at the University of Oxford. VGG-16 is characterized by its simplicity and uniform architecture, making it easy to understand and implement.

## 2 Literature survey

There are many works on tomato leaf detection, machine learning (Uma et al., 2016; Anusha and Geetha, 2022; Harakannanavara et al., 2022), deep learning (Haridasan et al., 2023; Sankareshwaran et al., 2023; Yakkundimath and Saunshi, 2023), optimization techniques, data mining (Das and Sengupta, 2020; Demilie, 2024), regression analysis, image analysis (Ganatra and Patel, 2020; Ngugi et al., 2021), and prediction techniques that are found in the literature. Mustafa et al. (2023) proposed a five-layer CNN model for detecting plant diseases using leaf images. A total of 20,000 images were used to train the model. This model detects the pepper bell plant leaf disease with better accuracy. The results are evaluated in terms of accuracy, precision, and recall, and F1-scores are computed. The model performs better than state-of-the-art models. Seetharaman et al. (Seetharaman and Mahendran, 2022) presented a region-based CNN model to detect a banana leaf disease using Gabor extraction. Images are preprocessed by histogram pixel localization with media filter. The segmentation part is done with region-based edge normalization. Feature extraction is performed using the novel method Gabor-based binary patterns with CNN. A region-based CNN helps in detecting the disease area. The results are evaluated and they perform better than CNN, DCNN, ICNN, and SVM models in terms of precision, recall, accuracy, and sensitivity.

Nerkar et al. (Nerkar and Talbar, 2021) proposed a method to detect leaf disease using a two-level nonintrusive method. This model combines generative adversarial network and reinforcement learning. Cross dataset learning is used. CNN is combined with GAN for better results. Re-enforcement learning retrains the GAN using confidence scores. Classification results are evaluated and results are higher than other models. Mukhopadhyay et al. (2021) proposed a non-dominated sorting genetic algorithm for tea leaf disease detection. Image clustering is the main idea of this model. PCA is used for feature reduction and multi-class SVM is used for disease detection. Five various datasets of tea leaf are used in the work. The proposed model provides better accuracy than traditional models.

Vallabhajosyula et al. (2022) proposed a transfer learning-based neural network for plant leaf disease detection. In this work, pre-trained models were used. The deep ensemble neural network is used along with pre-trained models. Transfer learning and data augmentation are used for parameter tuning. The results are evaluated and provide higher accuracy with lesser number of computations. Huang et al. (2023) discussed a tomato leaf disease detection model using the full convolutional neural network (FCN) with suitable normalization dual path networks. The FCN used to segment the target crop images and improve the dual path network model is used for feature extraction. The results are evaluated on the augmentation dataset and accuracy is better than other models.

Chouhan et al. (2021) proposed a model for leaf disease detection using the fuzzy-based function network. Initially, preprocessing is done and the scale-invariant feature transform method is used for feature extraction. The fuzzy-based function network is used for detecting the leaf disease. Training is done with the help of the firefly algorithm. The model results are evaluated in terms of accuracy and are higher than traditional models. He et al. (2023) presented a maize leaf disease detection model using machine vision. The batch normalization layer is appended with the convolution layer to fasten the convergence speed of the network. Cost function is developed to increase the detection accuracy. Four types of pre-trained CNN models are used for feature extraction network for training. The gradient descent algorithm is applied to optimize the model performance. The results are evaluated in terms of F1-score, recall rate, and accuracy.

Ruth et al. (2022) proposed a deep learning model for disease detection using the meta-heuristic algorithm. CNN is used for feature extraction. The optimal deep neural network is used for disease detection. A two-level weight optimization is used to increase the performance of the detection model. Two-level weight optimization is achieved using an improved butterfly optimization algorithm, where the genetic algorithm is used to improve the butterfly optimization algorithm. The results are evaluated in terms of sensitivity, accuracy, and specificity. The overall accuracy is higher than other traditional models. Andrushia et al. (Andrushia and Patricia, 2020) presented a leaf disease detection model using the artificial bee colony optimization algorithm. Initially, preprocessing is done by removing noises and background images. Shape, color, and texture are extracted as features and are sent to the support vector machine model for disease detection. The model results are better in terms of recall, precision, and accuracy.

Abed et al. (2021) presented a novel deep learning model for bean leaf disease detection. This model contains two phases: detection and diagnosing. For detection, the U-Net architecture using the ResNet34 encoder is used. In the classification part, results are evaluated for five different deep learning models. The dataset contains 1,295 images of three classes such as healthy, bean rust, and angular leaf spot. The results are evaluated in terms of sensitivity, specificity, precision, F1-score, and area under the curve (AUC). Pandey et al. (Pandey and Jain, 2022) proposed a deep attention residual network using an opposition-based symbiotic organisms search algorithm. In this model, residual learning blocks are used with the attention learning mechanism for feature extraction. A new CNN model, AResNet-50, is designed for disease detection. The opposition-based symbiotic organisms search algorithm is used to tune the parameters of the model. Plants like citrus, guava, eggplant, and mango leaves are considered for the experimental analysis. The results of the model are evaluated in terms of accuracy, and they are better than those of the existing models such as AlexNet, ResNet-50, VGG-16, and VGG-19. Zhao et al. (2020) proposed a multi-context fusion network model for crop disease detection. In this model, standard CNN is used to extract visual features from 50,000 crop disease samples. Contextual features are collected from image acquisition sensors. A deep, fully connected network is proposed by combining contextual features and visual features to detect the leaf disease. The model performance is evaluated in terms of accuracy, which is higher than state-of-the-art methods.

Wang et al. (2017) proposed a new technique for automatic estimation of plant disease severity using image analysis through the effective application of deep learning algorithms. Bracino et al. (2020) explained the development of a new hybrid model based on machine learning techniques for the accurate detection of health using disease classification. Ashwinkumar et al. (2022) proposed an automated plant leaf disease detection model using deep learning classification named optimal MobileNet, which is designed based on CNNs. Khan et al. (2019) developed one optimized method for disease detection using image segmentation and classification for identifying the apple diseases. The authors made the decisions by analyzing whether there is a strong correlation among the features and also using genetic algorithm for feature selection. Most of the works found in the literature on tomato leaf disease detection used the benchmark dataset, namely, the Plant Village dataset (Kaustubh, 2020).

Sanida et al. (2023) proposed a new methodology for the effective detection of tomato leaf diseases by identifying them using a two-stage transfer learning model. Pandiyaraju et al. (2023) proposed an optimal energy utilization technique for reducing the energy consumption via the agricultural sensors used in precision agriculture. These sensors have been connected to a WSN that performs energy optimization by using a multi-objective clustering and deep learning algorithm to reduce the energy consumption. In another related work, Pandiyaraju et al. (2020) developed an energy-efficient routing algorithm for WSNs using clustering of nodes. Moreover, the routing decision has been made in their work using intelligent fuzzy rules that were applied in precision agriculture. In the area of agriculture and gardening, Pandiaraju et al. (Pandiyaraju et al., 2017) proposed a rule-based intelligent roof control algorithm for effective water conservation without affecting the agricultural yield with respect to smart terrace gardening. Such a model can be enhanced to detect the leaf diseases for providing better yield with minimum water.

Shoaib et al. (2023) presented a review of deep learning classification algorithms that have been used in the detection of plant leaf diseases. Santhosh et al. (2014) proposed a farmer advisory system using intelligent rules based on machine learning classifier. Jabez Christopher et al. (Jabez et al., 2015) proposed an optimized classification model that uses rules based on knowledge mining with swarm optimization for providing effective disease diagnosis. Gadade et al. (Gadade and Kirange, 2022) proposed an intelligent approach based on deep learning for the effective detection of tomato leaf diseases from leaf images that have captured with varying capturing conditions. Saeed et al. (2023) proposed one new smart detection methodology for the accurate detection of tomato leaf diseases by using transfer learning-based CNNs. Shoaib Muhammad et al. (Shoaib et al., 2022) proposed a new model for tomato leaf disease detection by using deep learning algorithms for performing both segmentation and classification of leaf images.

Sreedevi and Manike (2024) presented a new solution for identifying the tomato leaf disease based on classification using a modified recurrent neural network through severity computation. Prabhjot Kaur et al. (2024) carried out a performance analysis on the image segmentation models that are used to detect leaf diseases present in the tomato plants. Thai-Nghe et al. (Nguyen et al., 2023) presented a deep learning-based approach for the effective detection of tomato leaf diseases. Chang et al. (2024) developed one general-purpose edge-feature-guided model for the identification of plant diseases by enhancing the power of vision transformers. Li et al. (2023) presented a new lightweight vision transformer model based on shuffle CNNs for the effective diagnosis of leaf diseases in sugarcane plants. Thai et al. (2023) proposed a new vision transformer model designed for the accurate detection of cassava leaf diseases.

Yu et al. (2023) explained the use of inception convolutional vision transformers for the effective identification of plant diseases. Arshad et al. (2023) developed an end-to-end and hybrid model based on the deep learning framework for the accurate prediction of potato leaf diseases. Shiloah et al. (Elizabeth et al., 2012) proposed one new segmentation approach based on machine learning model for improving the diagnostic accuracy of detecting lung cancers from chest computed tomography images. Dhalia Sweetlin et al. (2016) proposed a patient-specific model for the effective segmentation of lung computed tomographic images. Singh and Misra (2017) proposed a machine learning-based model for the effective detection of plant leaf diseases by performing suitable image segmentation. Agarwal et al. (2020) developed a new system for tomato leaf disease detection by applying the CNN classifier.

Chen et al. (2022) proposed the use of the AlexNet CNN model for the effective detection of tomato leaf diseases by performing accurate classification of tomato leaf images. Ganapathy et al. (2014) proposed an intelligent temporal pattern classification model by using fuzzy temporal rules with particle swarm optimization algorithm. Jaison et al. (Bennet et al., 2014) proposed a discrete wavelet transform-based feature extraction model along with one hybrid machine learning classification algorithm for performing effective microarray data analysis. Elgin Christo et al. (2019) proposed a new correlation-based ensemble feature selection algorithm that has been developed using bioinspired optimization algorithms integrated with a backpropagation neural network-based classifier.

Thangaraj et al. (2021) proposed an automated tomato leaf disease classification algorithm by using a transfer learning-based deep CNN classifier. Al‐Gaashani et al. (Al-gaashani et al., 2022) proposed a new model for tomato leaf disease classification by the application of transfer learning with feature concatenation. Han et al. (2017) proposed a new weighted gradient-enhanced classification model not only to provide high-dimensional surrogate modeling but also to perform design optimization. Wu et al. (2021) proposed a new distributed optimization method that uses weighted gradients for solving the economic dispatch problem pertaining to the multi-microgrid systems. Abouelmagd et al. (2024) developed an optimized capsule neural network for the effective classification of tomato leaf diseases. Other approaches that are used in the detection of leaf diseases include those with deep learning and also with explainable AI (Rakesh and Indiramma, 2022; Bhandari et al., 2023; Debnath et al., 2023; Nahiduzzaman et al., 2023).

Despite the presence of all these related work in the literature, most of the segmentation and classification algorithms use a machine learning approach for classification. Therefore, it is necessary to employ either manual work or additional classification algorithms for performing feature extraction and feature selection. Moreover, the time series data are not analyzed by giving higher importance to the most recent data by the application of temporal constraints. The convergence of the existing deep learning algorithm employed in the detection of tomato leaf diseases is not supported by an optimization algorithm. Finally, ensemble-based classification algorithms are not employed in the classification process to enhance the detection accuracy. In order to handle all these limitations that are present in the existing systems developed for accurate tomato leaf disease detection, a new ensemble classification model is proposed in this paper that uses an EMA function with temporal constraints, and it is supported by an EWGO along with fine-tuned VGG-16 and NASNet mobile training methods for enhancing the classification accuracy that can increase the detection accuracy with respect to the detection of tomato leaf diseases.

## 3 Proposed work

### 3.1 Method

The data that show the features are initially analyzed using histogram plots and pie charts for better visualization of the data statistics to check for data imbalance among different classes. It has been concluded via complete exploration that there is no data imbalance and that the features of the images have been completely studied.

Next, the images are preprocessed in order to enhance the learning ability of our deep learning models. A median filter is applied on the image to remove noise to improve image quality. Redundant parts of the image that do not contribute to the model’s learning process are also removed. Furthermore, the α and β factors in our images are adjusted in order to modify the brightness and contrast, thereby making the region of interest more prominent. The images are finally normalized to have pixel values ranging from 0 to 1, and the data are augmented to ensure a wider scale of learning by the model.

For the initial part of feature extraction, the VGG-16 transfer learning model undergoes fine-tuning by unfreezing its last five layers, enabling to adapt the model that originally contained ImageNet’s weights to the specified dataset. By employing the use of Global Average Pooling to pool the CNN layers’ features, the data are then passed into two fully connected layers ultimately leading to the output layer. The optimization of the model is achieved using the Adam optimizer with a learning rate of 0.0001, and evaluation metrics such as the F1-score, AUC score, precision, and recall are applied.

The NASNet mobile transfer learning model is employed with ImageNet weights for the next part. A flattened layer is then used to transform the outputs from the CNN layers into a one-dimensional tensor that facilitates the passage through three fully connected layers that ultimately reach the output layer. The optimization of the model is once again achieved using the Adam optimizer with a learning rate of 0.0001, and evaluation metrics such as the F1-score, AUC score, precision, and recall are applied.

The extracted features obtained from the two transfer learning models are now taken and passed on as parameters to a custom ensemble layer that incorporates EMA function that emphasizes the recent data points with greater weights. The resulting ensemble model shows an optimized learning curve by adopting the adaptive rate of learning, which is achieved by using a custom EWGO that modifies the learning rate based on custom ensemble weight suitable for our custom ensemble model.

### 3.2 Dataset

This research utilizes the dataset (Kaustubh, 2020) that consists of a collection of tomato leaf images, each belonging to one of nine distinct categories, representing various leaf diseases or a healthy state (no disease). The dataset encompasses a total of 10,000 images designated for training and an additional 1,000 images reserved for testing. To facilitate model development and evaluation, we partitioned the training dataset into a 75%–25% split, resulting in 7,500 images allocated for training and 2,500 images for validation, and the entire additional 1,000 images were reserved for the test set.

This dataset serves as the foundation for the development of the proposed model, which aims to enhance the classification of tomato leaf diseases.

### 3.3 Preprocessing

The following are the steps involved in preprocessing:

- Median filter
- Image cropping
- Brightness and contrast adjustments
- Normalization

#### 3.3.1 Median filter

The first step of data preprocessing utilizes a median filter, which is a non-linear digital image filtering technique that runs through the signal as one entry after another by replacing the entry value by the median of the neighboring entry values, which depends on the window size, resulting in the removal of the salt-and-pepper noise in an image. In this case, a window size of 3 has been chosen for preprocessing the image.

This median filter is represented mathematically as shown in Equation (1):


(1) g(x,y)=Med(f(x,y))

where *f*(*x,y*) is the window array and *g*(*x,y*) is the median value of the window array. The steps for the median filter are shown in **Algorithm 1**.

Algorithm 1 **Median filter.**

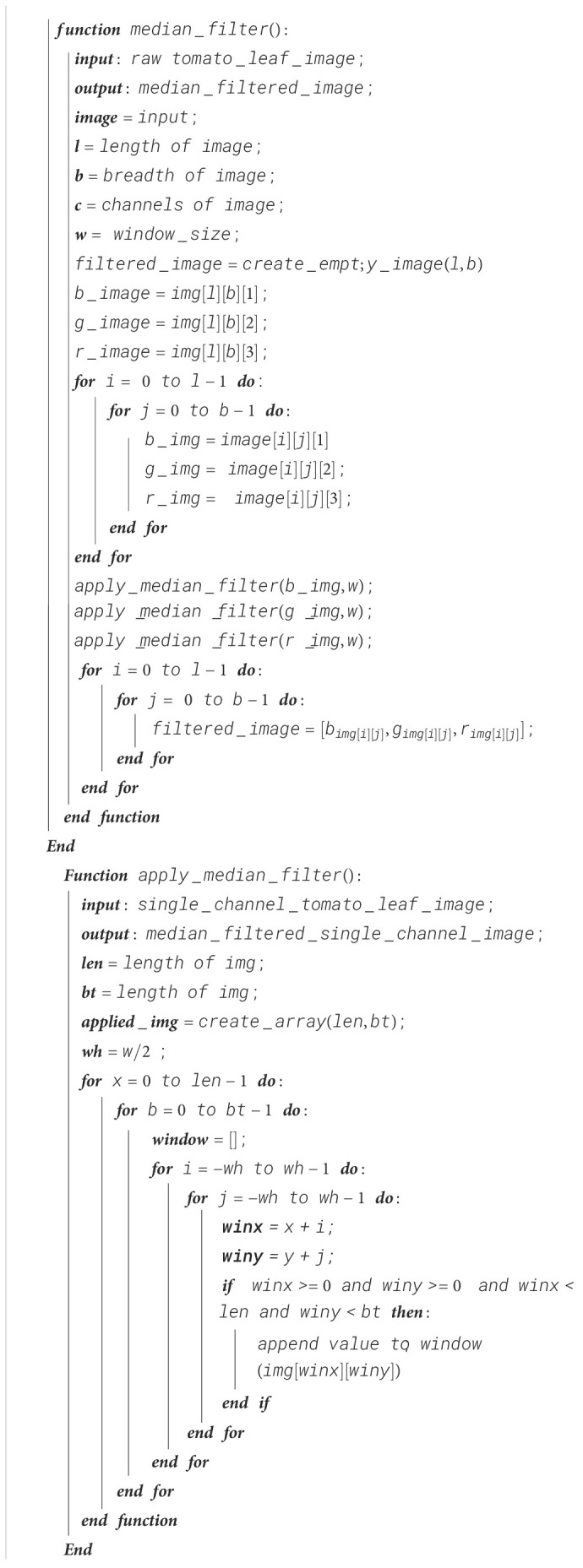

#### 3.3.2 Image cropping

Since the outer areas of the image are not helpful with the tomato disease detection, the size of the image is reduced by 10 pixels on each side, thus reducing the image size from 256 × 256 to 236 × 236 by removing the areas where there are no significant features for disease detection. The steps for image cropping are shown in **Algorithm 2**.

Algorithm 2 **Image cropping.**

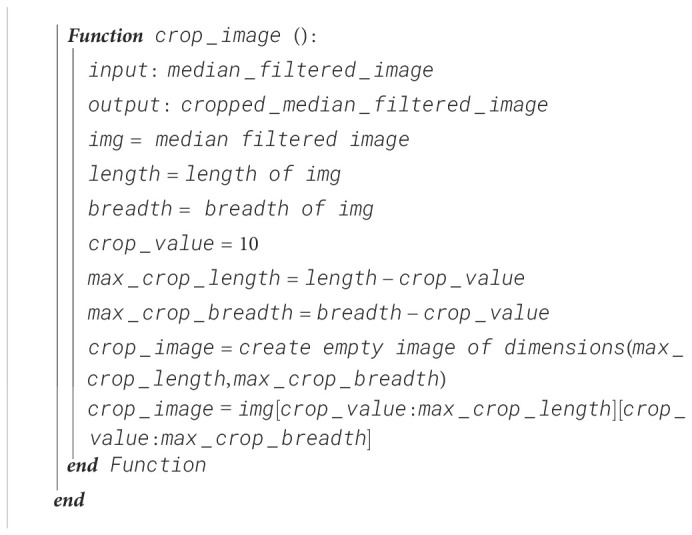

#### 3.3.3 Brightness and contrast enhancements of images

For better-quality images and improved ability of the CNN to identify the region of interest, its brightness is reduced and the contrast of the image is increased. This mitigates overexposure of the images, allowing the CNN to extract the features in the region of interest easily due to better visibility.

Brightness and contrast enhancement can be represented mathematically as shown in Equation (2):


(2) g(i,j)=αf(i,j)+β

where *α* is the contrast factor and *β* is the brightness factor. *f*(*i*,*j*) represents the pixel of the input image, which is the cropped image, while *g*(*i*,*j*) is the output image where the image’s brightness and contrast are adjusted using *α* and *β*. The procedure for brightness and contrast enhancements is shown in **Algorithm 3**.

Algorithm 3 **Brightness and contrast enhancement.**

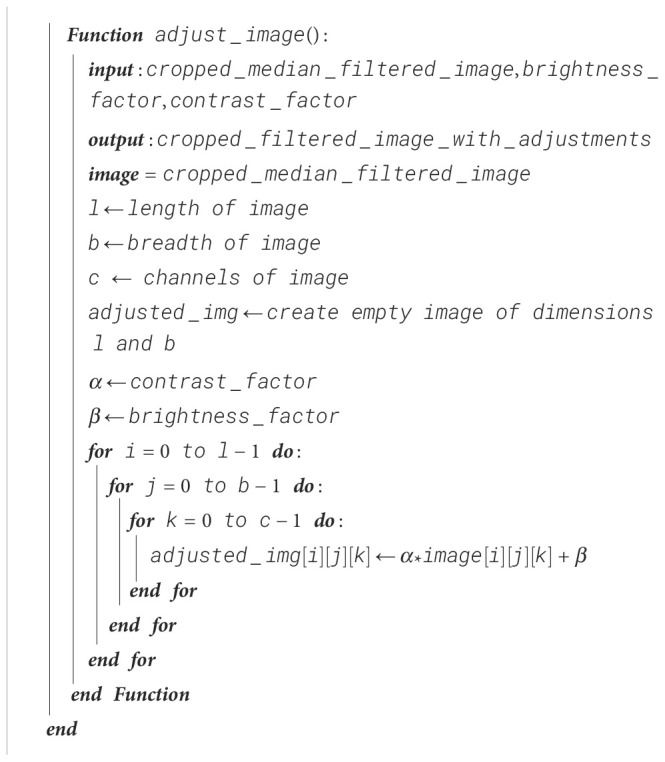

#### 3.3.4 Image normalization

For better weight initialization and to maintain consistency in the pixel range of the input, the image is normalized so that all pixel values are confined to the interval [0, 1]. Due to this, the deep learning model’s convergence is enhanced with the range reduction from 255 to 1 by dividing each pixel value by 255. This process also improves the learning rate of our proposed model and the stability of the model during training. The procedure for image normalization is shown in **Algorithm 4**.

Algorithm 4 **Image normalization.**

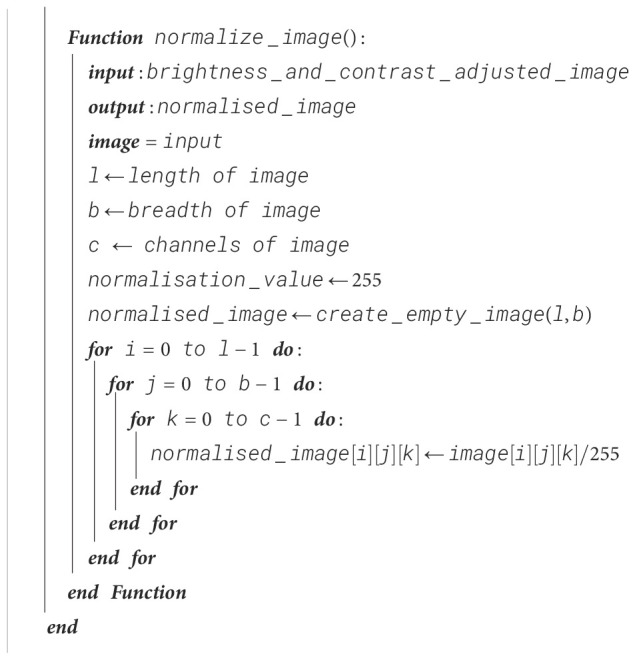

### 3.4 Feature extraction and classification

Upon successful completion of preprocessing, the tomato leaf images are subjected to appropriate feature extraction and thereby will be classified using the deep learning model. This, in turn, will support not only the identification of diseases in the leaves but also the severity. The deep learning model used is the VGG-16 fine-tuned model. In addition, a CNN model, namely, NASNet, is also employed for the leaf’s disease identification.

Later, an ensemble model consisting of five ensemble blocks and a final output block is used with the input layer being received from the output of the VGG-16 fine-tuned model and the NASNet model as a list. Furthermore, the results are improved for an enhanced performance with the aid of an EMA-based approach and optimized with an EWGO.

#### 3.4.1 VGG-16 fine-tuned model

The last five layers of the VGG-16 model are unfrozen and the weighs of these layers are updated with the data to fine-tune the model. The optimizers do not modify the parameters of the remaining layers, which remain frozen, thereby preserving the weights.

This model, which is made up of five different blocks, is composed of convolution layers with rectified linear unit (ReLU) activation and a max pooling layer, a global average pooling layer, dense layers, batch normalization layers, and an output dense layer with softmax activation. The preprocessed image of size 236 × 236 × 3 is taken as an input into the model, first entering block 1.

Block 1 consists of two convolution layers and a max pooling layer. Each convolution layer consists of 64 filters, each of size 3 × 3. Each layer also has a ReLU activation layer that brings in non-linearity once the feature extraction is done by that layer. The first convolution layer receives the input as 236 × 236 × 3, and the first convolution layer produces the output of shape 236 × 236 × 64 after the activation function. The second convolution layer takes the input as the output of the first convolution layer and performs feature extraction and ReLU activation without making any changes in the shape of the data. Once the output data are produced by the second convolution layer, the max pooling layer that has a filter size of 2 × 2 reduces the size from 236 × 236 × 64 to 118 × 118 × 64, which sends the output to block 2.

Block 2, just like block 1, consists of two convolution layers where each layer has a ReLU activation function and a max pooling layer. The only difference is that the input received by the first convolution layer of this block will be of size 118 × 118 × 64. At the end of the second convolution, the output will be of size 118 × 118 × 128 since the number of filters in the convolution layers of the second block is 128. The max pooling layer reduces the size of the data from 118 × 118 × 128 to 59 × 59 × 128.

Block 3, unlike the previous two blocks, has three convolution layers where each layer has a ReLU activation function and a max pooling layer. The functionality of the block remains the same with the difference here being the presence of a third convolutional layer and the presence of 256 filters in each convolution layer. The first convolution layer receives the input of size 59 × 59 × 128 from the max pooling layer of block 2 and produces an output of size 59 × 59 × 256, which is preserved in the second and third convolution layer. The max pooling layer reduces the size of the data to 29 × 29 × 256.

Blocks 4 and 5 are similar to block 3 with the only difference being all the convolution layers present in blocks 4 and 5 have 512 filters. The input received by the first layer of block 4 will be of dimension 29 × 29 × 256 and the output after the third convolution layer will be of size 29 × 29 × 512, which, in turn, is reduced to 14 × 14 × 512 by the max pooling layer. In case of block 5, the input received by the first convolution layer will be of size 14 × 14 × 512 and the output is preserved even after the third convolution layer. The max pooling layer in block 5 reduces its size from 14 × 14 × 512 to 7 × 7 × 512.

The global average pooling layer takes the output of block 5 as input, which down-samples the multi-dimensional data into single-dimensional data by finding the average of each feature map where the filter is of size 2 × 2, resulting in the reduction of data size from 7 × 7 × 512 to 1 × 1 × 512. After this down-sampling, two dense layers with ReLU activation composed of 128 and 32 neurons, respectively, transform the output obtained by extracting the features of the preceding layers into data, which are suitable for classification. Finally, the output layer, i.e., dense layer with softmax activation, is used to perform multiclass classification. The steps for VGG-16 fine-tuned model is shown in **Algorithm 5**.

Algorithm 5 **Tomato leaf classification—fine-tuned VGG-16 training.**

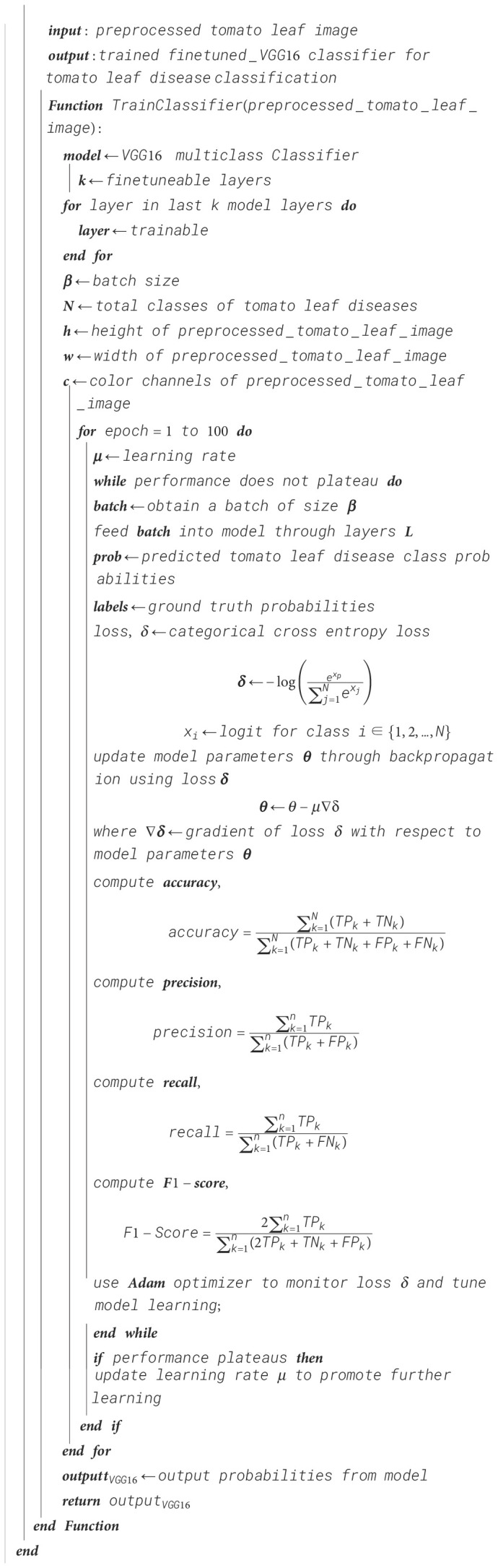

Algorithm 6 **Tomato leaf classification—NASNet training.**

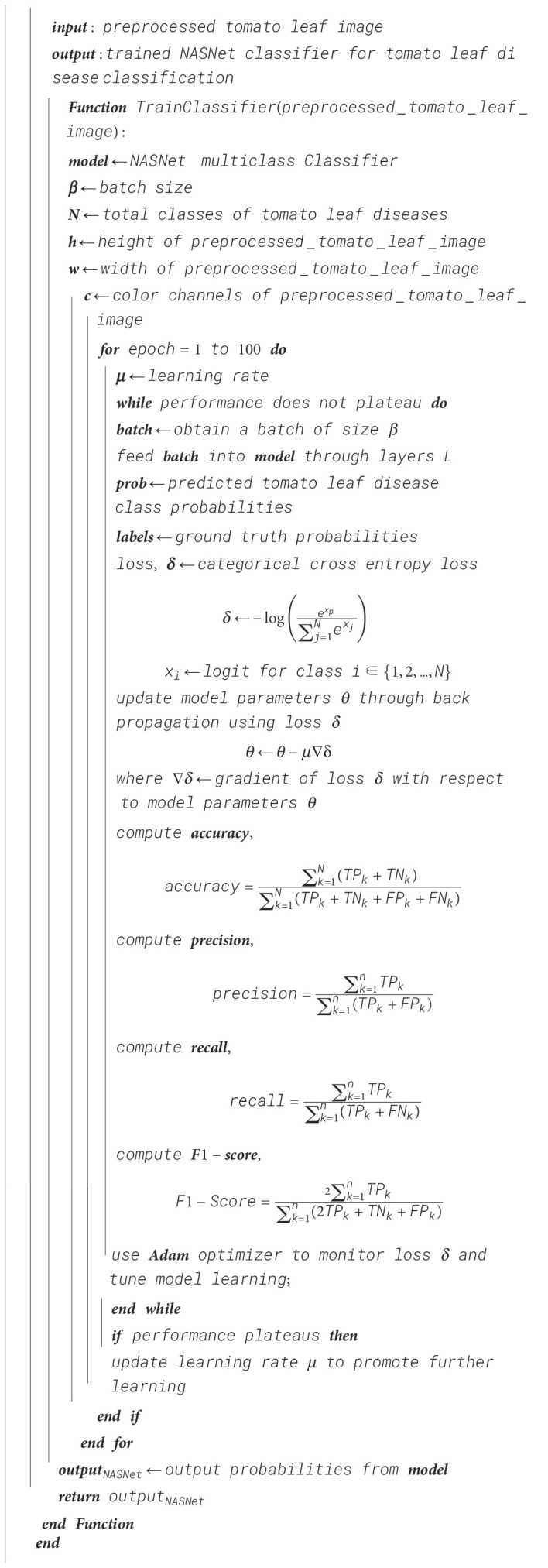

#### 3.4.2 NASNet

NASNet is a deep learning architecture where an optimal neural architecture is searched automatically by using the Neural Architecture Search (NAS) method. For the best performance on a specific task, the design of the neural network’s topology is automated using the NAS process.

The NAS algorithm can be generalized as an algorithm that searches for the best algorithm to perform a certain task. It involves three different components, namely, the search space, performance estimation strategy, and search strategy. The search space encompasses all the potential architectures that can be looked for within the neural network’s subspace. It can be categorized into two primary types: the global search space and the cell-based search space. The global search space offers a high degree of flexibility, accommodating a wide range of architecture due to its ample operation arrangement options. In contrast, the cell-based search space is characterized by recurring fixed structures in effective, manually designed architectures, leading to the assembly of smaller cells into larger architectural structures.

Without construction or training of a possible neural network, the performance is evaluated using the performance estimation strategy, which returns a number or an accuracy value of the possible model architecture, which the NASNet predicts as a possible solution. Different search strategies such as grid search, random search, gradient-based search, evolutionary algorithm, and reinforcement learning can be used to identify the best architectures and avoid bad ones before estimating performance. The steps for NASNet training is shown in **Algorithm 6**.

#### 3.4.3 Ensemble model

The ensemble consists of five ensemble blocks and a final output block. The input layer receives the output of the VGG-16 fine-tuned model and the NASNet model as a list. This input is then passed through the five ensemble blocks, finally reaching the output layer. Each ensemble block is composed of a fully connected layer, a reshape layer, two convolutional layers, a batch normalization layer, ReLU activation, an ensemble layer, and a max pooling layer.

The ensemble process in the ensemble layer is carried out based on effective moving average. This layer has two parameters, namely, the decay rate, which is responsible for reducing the effective moving average, and the update rate, which ensures that for every update rate iteration, the weights in the ensemble layer will be modified with the help of the effective moving average.

The output layer is responsible for classification.

The effective moving average is represented mathematically as shown in Equation (3):


(3) $$EMA_{updated}=EMA+\left(Pred_{NASnet}-Pred_{VGG16FT}\right)*\delta$$

where EMA_updated_ is the updated effective moving average; EMA denotes the effective moving average before the update operation; Pred_NASNet_ and Pred_VGG16FT_ are the predictions of NASNet and the VGG-16 fine-tuned model, respectively; and *δ* is the decay rate, which is taken as 0.8 in this case.

The predictions of both models are taken as input. Initially, the prediction of the VGG-16 fine-tuned model was taken as the effective moving average, which is then updated with the help of the above mathematical expression. The update rate ensures that the weights are modified only after a certain number of iterations, which is two in this case. Therefore, for every second iteration, the weights are modified by reshaping the effective moving average tensor for every weight tensor. The reshaped tensor is updated into the weight tensor as the new weight tensor for the next two iterations. The procedure for Ensemble classifier training using EMA is shown in **Algorithm 7** and procedure for exponential moving average-based ensemble weight update in a custom ensemble layer is shown in **Algorithm 8**.

Algorithm 7 **Ensemble classifier training using EMA for tomato leaf disease classification.**

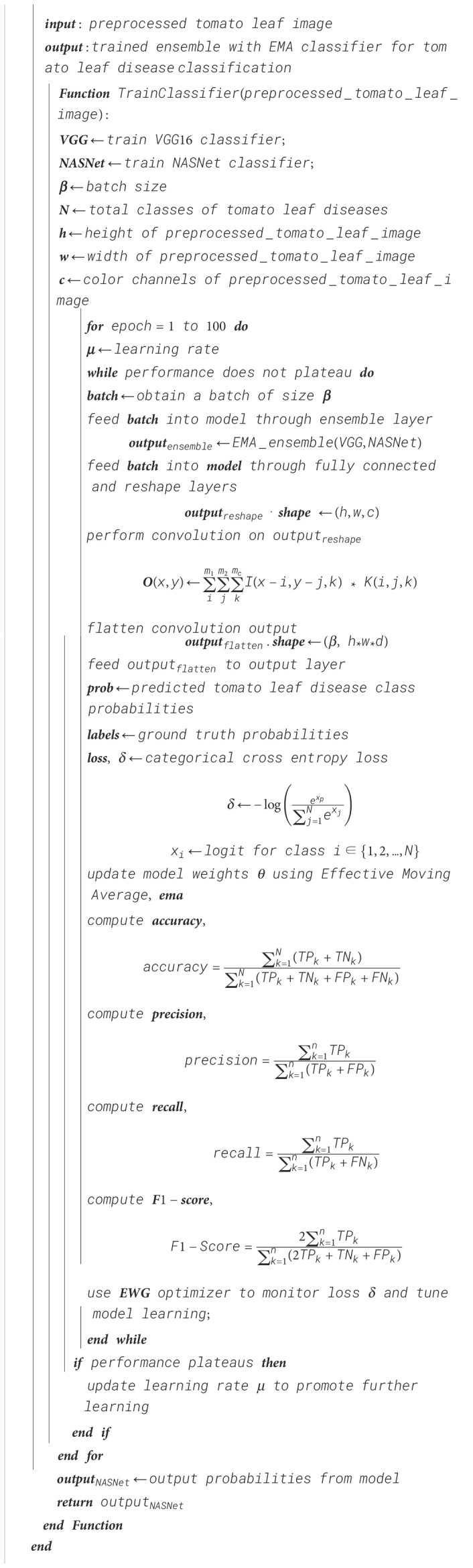

Algorithm 8 **Exponential moving average-based ensemble weight update in a custom ensemble layer.**

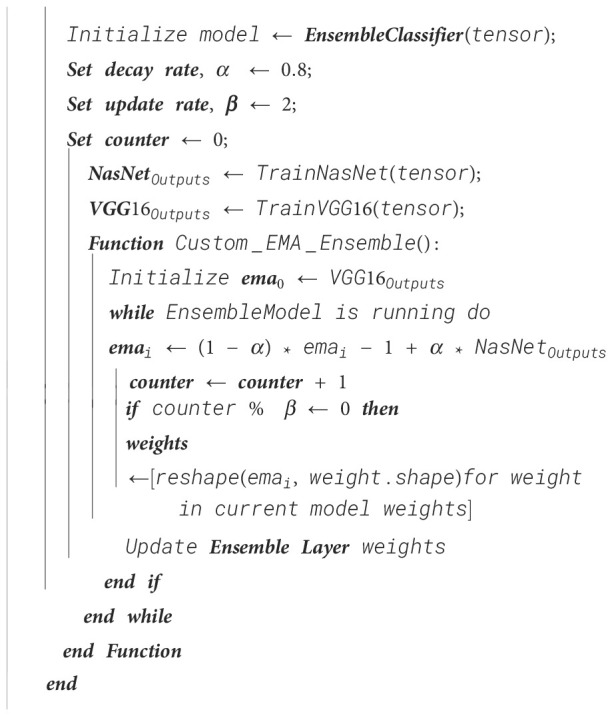

#### 3.4.4 Layer information during feature extraction

There are a total of 12 layers used during feature extraction as enumerated below.

##### 3.4.4.1 *(i)* Convolutional layer

The convolutional layer is the most important layer used in CNNs, which is responsible for extracting features from the input with the use of filters or kernels. The kernel is a matrix consisting of a set of learnable parameters. The convolution process can be defined as the conversion of pixels in its receptive field into a single pixel. This operation is performed as the dot product between the kernel matrix and another matrix, which is the receptive field restricted to a certain portion. Hence, in the input image that is composed of three color channels, the kernel carries out the convolution operation in all the three channels, although the height and width will be spatially small. The kernel slides across the height and width of the receptive region of the image. This sliding size is called a stride. The result is a production of a two-dimensional representation of the kernel at each spatial position of the image. The convolution operation results in a feature map as output, which can be represented mathematically as shown in Equation (4):


(4) $$O\left(x,y\right)=\sum_{i=-\infty }^{\infty }\sum_{j=-\infty }^{\infty }I\left(x-i,y-j\right)*K\left(i,j\right)$$

where *O*(*x,y*) represents the value in the output feature map in the position (*x, y*) and *I*(*x−I,y−j*) represents the pixel value in the input at position (*x−i,y−i*). *K*(*i,j*) represents the value of the kernel at position (*i,j*).

##### 3.4.4.2 (ii) Depthwise separable convolutional layer

Depthwise separable convolution handles both the spatial and depth dimensions. Here, the kernels cannot be factored into smaller units. This process is split into two steps:

- Depthwise convolution: a single convolution filter is applied on each input channel.
- Pointwise convolution: it involves the usage of a 1 × 1 filter that iterates through every single point of the input.

This kernel has a depth equal to the number of channels that the input has. The usage of a depthwise separable convolution layer reduces the number of parameters compared to the standard convolution layer.

##### 3.4.4.3 *(iii)* Max pooling layer

The max pooling layer is one of the largely used layers in CNNs, normally found after the convolutional layer. Its purpose is to reduce the spatial dimensions (length and breadth in this case) of the input feature map resulting from the preceding convolution layer. The feature map is taken by the layer as input, which applies the max pooling operation where a window slides through the feature map the window content with the maximum value in the window, thus down-sampling the feature map. Providing a stride value lets the CNN know the number of pixels to move while sliding through that particular layer. The max pooling layer can be mathematically represented as shown in Equation (5):


(5) $$O\left(x,y\right)=max_{i=0}^{k-1}max_{j=0}^{k-1}I\left(x\cdot s+i,\;y\cdot s+j\right)$$

where *O*(*x,y*) is the value in the output feature map at point (*x,y*), *s* is the stride value, and *I*(*x·s+i,y·s+j*) is the value in the input feature map at position (*x·s+i,y·s+j*), and *k* is the size of the pooling window.

##### 3.4.4.4 *(iv)* Average pooling layer

The purpose of using the average pooling layer is to reduce the spatial dimensions such as the length and depth of the feature map just like the max pooling function, but the difference here is that down-sampling is performed by transforming the window into a single value, which is the average of the values present in it. This returns a smoother feature map compared to the max pooling layer, which returns a feature map focusing on prominent features. The average pooling layer can be mathematically represented as shown in Equation (6):


(6) $$Y\left[i,j,c\right]=\frac{1}{k_{h}*k_{w}}\;\sum_{p=0}^{k_{h}-1}\sum_{q=0}^{k_{w}-1}X\left[i*s_{h}+p\;,\;j*s_{w}+q,c\right]$$

where *Y* is the output after the pooling function, *X* is the input feature map, $k_{h}$ is the height of the feature map, and *k_w_* is the width of the feature map. $s_{w}$ and $s_{h}$ are the stride values for height and width while sliding through the input feature map.

##### 3.4.4.5 *(v)* Concatenation layer

The concatenation layer concatenates the inputs having the same size in all dimensions except the concatenation dimension, received by the layer along a specified dimension. This layer is used whenever we want to merge the information from different parts of the network or data modalities. The concatenation operation takes place by combining multiple input tensors by stacking them along the specified axis, resulting in a single tensor with an increase in size. The layer is mathematically expressed as shown in Equation (7):


(7) $$O\left[i,j,c\right]=\left\{ \begin{array}{l} \;A\left[i,j,c\right]\;if\;0\leq c<C_{1}\\ B\left[i,j,c-C_{1}\right]\;if\;C_{1}\leq c\leq C_{1}+C_{2} \end{array}\right.$$

where *O* is the output, *A* is the first input tensor with *C*_1_ channels and *B* is the second input tensor with *C*_2_ channels for the concatenation layer, *i* represents the height dimension and ranges from 0 to *H*, *j* represents the width dimension and ranges from *j* to *W*, and *c* represents the channels and ranges from 0 to *C*_1_+*C*_2_.

##### 3.4.4.6 *(vi)* Addition layer

This layer adds inputs from multiple neural network element-wise. This operation can be performed when the input tensors have the same shape. This is done so that the information flows seamlessly through the network just by the addition of the output of one layer to the output of the previous layer. This layer is mathematically represented as shown in Equation (8):


(8) O[i,j,c]=A[i,j,c]+B[i,j,c]

where *O* is the output, *A* is the first input tensor and *B* is the second input tensor for the addition layer, *i* represents the height dimension and ranges from 0 to *H*, *j* represents the width dimension and ranges from *j* to *W*, and *c* represents the channels and ranges from 0 to *C*.

##### 3.4.4.7 *(vii)* Batch normalization layer

This layer helps in making neural networks faster and more stable by performing standardization and normalization operations in the feature map that is provided as input to the layer. The normalization process is carried out in two steps:

- Normalization
- Rescaling and offsetting

Before performing normalization, the data are fed into the layer in the form of mini batches. The mean and standard deviations of these mini batches can be found using the following equations shown in Equations (9, 10):


(9) $$\mu =\frac{1}{m}\sum_{i=1}^{m}x_{i}$$

and


(10) $$\sigma^{2}=\frac{1}{m}\sum_{i=1}^{m}{\left(x_{i}-\mu \right)}^{2}$$

where *µ* and *σ* are the mean of the values in the *i*th value in the mini-batch *x* of size *m*.

The main purpose of normalization is to transform the data to have a mean equal to 0 and standard deviation equal to 1, which is carried out using the expression as shown in Equation (11):


(11) $$x_{i\left(norm\right)}=\frac{x_{i}-µ}{\sigma +\epsilon }$$

Two learnable parameters *γ* and *β* are used for rescaling and offsetting, respectively, thereby normalizing each batch accurately. This is represented using the expression shown in Equation (12):


(12) $$x_{i}=\gamma x_{i\left(norm\right)}+\beta$$

where $x_{i}$ is the *i*th value of mini batch *x* and $x_{i\left(norm\right)}$ is the normalized *i*th value of mini batch *x.*

##### 3.4.4.8 *(viii)* Dropout layer

The dropout layer acts as a mask to nullify some of the neurons’ contributions towards the next layer while the rest of the neurons remain unmodified. It aims to prevent overfitting, avoid dependency on a specific neuron during training, and ensure better generalization from the model. The neurons are nullified using a probability for random exclusion such that they behave like they are not part of the architecture. The layer can be mathematically represented as shown in Equations (13, 14):


(13) O=X*M during training

and


(14) O=X*(1−p)during testing

where *O* is the output, *X* is the input, and *p* is the probability, and it is scaled to a factor (1 − *p*) during output since the dropout will be turned off during the testing phase. *M* is a binary mask with the shape same as *X* and each element of *M* is set as 0 or 1 depending on *p*.

##### 3.4.4.9 *(ix)* Global average pooling layer

The global average pooling layer is a pooling layer that performs down-sampling. Unlike the usual pooling layer, the global pooling layer condenses the feature maps into a one-dimensional mapping that can easily be read by the single dense classification layer. The mathematical representation is as shown in Equation (15):


(15) $$O=\frac{1}{H*W}*\sum_{i=0}^{H}\sum_{j=0}^{W}\left(F\left[i,\;j\right]\right)$$

##### 3.4.4.10 *(x)* Flatten layer

This layer performs the flattening operation that reshapes the input received into a single-dimensional feature vector without affecting the batch. It is done to allow the fully connected layers to operate on the multi-dimensional feature maps since the fully connected layers can only be trained with single-dimensional feature vectors.

##### 3.4.4.11 *(xi)* Fully connected layer

The fully connected layer or simply the dense layer is a CNN layer where all the neurons or nodes in one layer is connected to every node to the next layer. This layer works with activation functions such as the ReLU during feature extraction and softmax during multiclass classification. It is represented as a mathematical function as shown in Equation (16):


(16) O=f(W*X+b)

where *X* is the input, *O* is the output, *W* is the weight matrix, *b* is the bias vector, and *f* is the activation layer, which would be ReLU in case of feature extraction and softmax in case of classification.

##### 3.4.4.12 *(xii)* ReLU activation layer

The ReLU is a piecewise linear function used to introduce non-linearity into the feature map obtained as output before the activation function is applied. The ReLU function works by applying a simple thresholding operation where the positive values remain the same while the negative values become zero. The ReLU activation function can be expressed mathematically as shown in Equation (17)


(17) f=max(x,0)

where *x* is the input given into the function and *f* is the output obtained.

#### 3.4.5 Classification

##### 3.4.5.1 *(i)* Softmax activation

The softmax activation function is responsible for the multi-class classification of the vector obtained from the convolution layers after the feature extraction phase in the output layer. It works by calculating the exponent of each entry in the vector and dividing the value by the sum of all the exponents in the vector as shown in Equation (18).


(18) $$softmax\left(x_{i}\right)=\frac{e^{x_{i}}}{\sum_{j=1}^{N}e^{x_{j}}}$$

where *x* is the input vector and *i* is the *i*th entry in the input vector with *N* entries. The denominator of the softmax activation is the sum of the exponents of the entries. This is done for the conversion of *N* real number entries into a probability distribution of *N* possible outcomes.

##### 3.4.5.2 *(ii)* Categorical cross-entropy loss function

This loss function (also known as softmax loss) is used with a CNN to provide an output for the probability of each image over *N* different classes. This function is a combination of softmax activation and the cross-entropy loss function and is thus useful during multi-class classification. Its use allows the comparison of the target and predicted values by the CNN model as an output, thereby measuring the modeling efficiency of the training data by the CNN. The objective of this loss function is to calculate the difference between the ground truth and predicted class distribution. Techniques like gradient descent are used to adjust the weights and biases for minimalization of this loss, thereby improving the predictions. The categorical cross-entropy loss function is written as the negation of logarithmic function of the softmax function as shown in Equation (19):


(19) $$CE=-\log\left(\frac{e^{x_{p}}}{\sum_{j}^{N}e^{x_{j}}}\right)$$

where CE is the cross-entropy loss, *x_p_* is the positive class’ CNN score, *N* is the number of classes for classification, and *x_j_* is the *j*th class’ score.

To backpropagate through the network and optimize the defined loss function resulting in tuning the net parameters, the loss’ gradient is calculated with respect to the CNN’s output neurons given by the gradient of the cross-entropy loss with respect to each CNN’s class score. The derivatives are represented mathematically as shown in Equations (20, 21):

Derivative with respect to positive class:


(20) $$\frac{\partial }{\partial x_{p}}\left(-\log\left(\frac{e^{x_{p}}}{\sum_{j}^{N}e^{x_{j}}}\right)\right)=\frac{e^{x_{p}}}{\sum_{j}^{N}e^{x_{j}}}-1$$

Derivative with respect to negative class:


(21) $$\frac{\partial }{\partial x_{n}}\left(-\log\left(\frac{e^{x_{p}}}{\sum_{j}^{N}e^{x_{j}}}\right)\right)=\frac{e^{x_{n}}}{\sum_{j}^{N}e^{x_{j}}}$$

where *x_n_* is the score of any negative class in *N* other than *N*_p_, which consists of the positive classes.

#### 3.4.6 Optimizer

##### 3.4.6.1 *(i)* Adam optimizer

The Adam optimizer is an extension of the stochastic gradient descent (SGD) algorithm based on adaptive moment estimation, which takes advantage of two principles, namely, the momentum and root mean square propagation (RMSprop). The momentum technique is used to accelerate convergence in gradient descent by adding the fraction of the previous gradient update with the current update, reducing the oscillations. The convergence process speeds up along shadow dimensions, which assists optimization. RMSprop adapts the learning rate for each parameter individually by maintaining a moving average of squared gradients. This helps in scaling learning rates and making the optimization process more robust. With the help of these two methods, the following are obtained as shown in Equations (22, 23):


(22) $$m_{t}=\beta_{1}m_{t_{-1}}+\left(1-\beta_{1}\right)\left[\frac{\delta L}{\delta W_{t}}\right]$$

(23) $$v_{t}=\beta_{2}v_{t_{-1}}+\left(1-\beta_{2}\right){\left[\frac{\delta L}{\delta W_{t}}\right]}^{2}$$

where *m_t_* is the estimate of the first-order moment, which is the aggregate of gradients at time *t*, *v_t_* is the estimate of the second-order moment, which is the sum of the squares of the past gradients at time *t*, *β*_1_ is the decay rate of average of gradient in the momentum principle, and *β*_2_ is the decay rate of average of gradient in the RMSprop principle. The moment estimates *m_t_* and *v_t_* can be called the weight parameters.

In the Adam optimizer, the bias-corrected weights are considered such that the weight parameters will not be biased towards 0. The bias-corrected weight parameters are as shown in Equations (24, 25):


(24) $$\hat{m_{t}}=\frac{m_{t}}{1-\beta_{1}^{t}}$$

(25) $$\hat{v_{t}}=\frac{v_{t}}{1-\beta_{2}^{t}}$$

These bias-corrected weight parameters are used in the general weight update equation as shown in Equation (26):


(26) $$w_{t+1}=w_{t}-\hat{m_{t}}\left(\frac{\alpha }{\sqrt{\hat{v_{t}}}+\epsilon }\right)$$

where *α* is the learning rate or the step size parameter and *ϵ* is a small positive constant to avoid division by 0.

##### 3.4.6.2 *(ii)* Enhanced weighted gradient optimizer

This is a modified Adam optimizer that accepts a custom weight as a parameter and incorporates the gradients multiplied by the custom weight into its operation. The custom weights are given as a parameter and are introduced into the gradients with the values being multiplied. The modified values are introduced into the Adam optimizer and then used in our ensemble model. The updated weight with the custom weight parameter before optimization is as shown in Equation (27):


(27) $$\omega =\gamma \cdot w_{t}$$

This updated weight *ω* is introduced to the weight update process as shown in Equation (28).


(28) $$w_{t+1}=\omega -\hat{m_{t}}\left(\frac{\alpha }{\sqrt{\hat{v_{t}}}+\epsilon }\right)$$

where *γ* is the custom weight parameter, *α* is the learning rate or the step size parameter, *ϵ* is a small positive constant to avoid division by 0, *w_t_* is the existing weight before the optimization process, and *w_t_*_+1_ is the updated weight after optimization. $\hat{m_{t}}$ and $\hat{v_{t}}$ are the bias-corrected weight parameters. The procedure for enhanced weighted gradient optimizer is shown in **Algorithm 9**.

Algorithm 9 **Enhanced weighted gradient optimizer.**

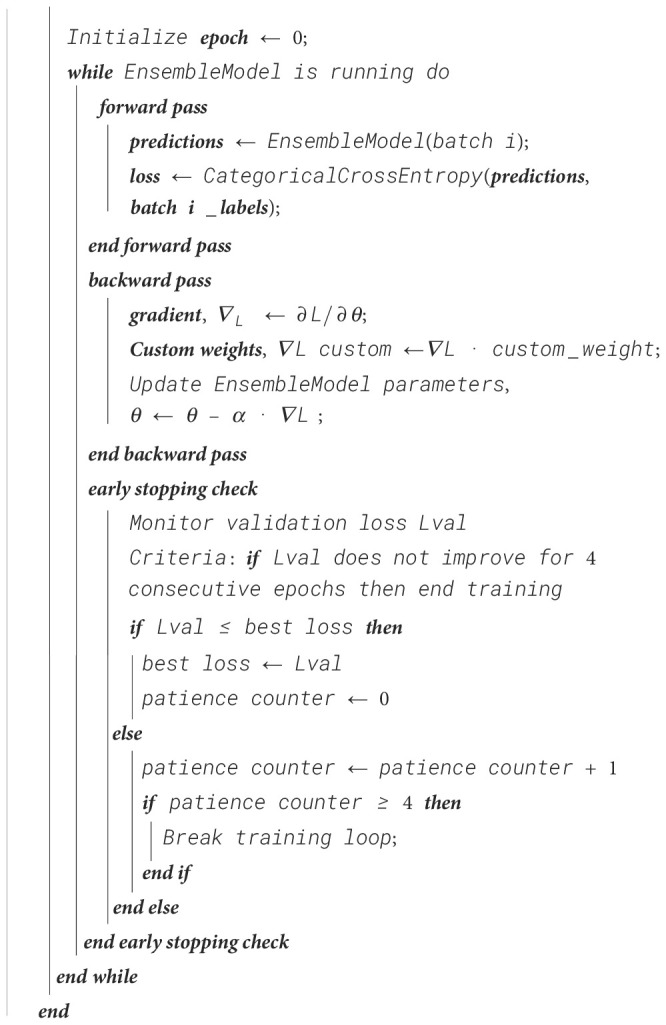

## 4 Results and discussion

In this paper, the focus of research starts by addressing a pressing issue in agriculture: the management of plant diseases, with a specific focus on tomato plants. Researchers have employed complex deep learning methodologies and machine learning models to tackle this challenge. This paper strives to revolutionize the ways to identify plant diseases, especially those affecting tomato plants, and manage them accordingly.

The study adopts data analysis and image preprocessing techniques to ensure that the dataset used is well-balanced and that the quality of the images is optimized for deep learning models. It uses methods such as median filtering, resized cropping, and brightness normalization to enhance the features derived from them. This meticulous attention to data quality and balance is crucial in developing a reliable disease classification system. To extract relevant features from the tomato leaf images, the research leverages two transfer learning models, VGG-16 and NASNet. Furthermore, these models are fine-tuned, allowing them to adapt to the specific characteristics of the dataset. This adaptability showcases the potential for pre-trained models to significantly improve classification accuracy when applied to particular datasets.

One of the key novelties is the incorporation of an ensemble model with an EMA function and an EWGO. This innovative approach optimizes the learning process, resulting in a more effective and accurate disease classification system. It stands as a promising method to enhance the performance of machine learning models in agriculture.

### 4.1 Performance metrics

**Figure 1:**
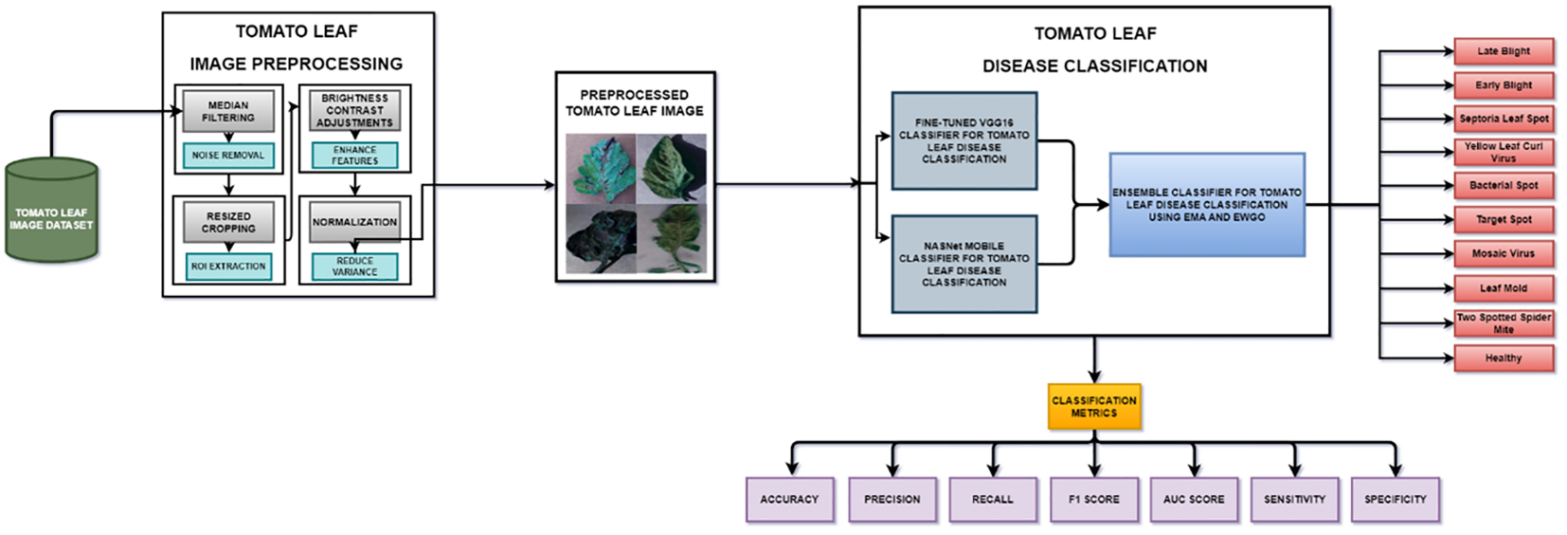
Overall proposed architecture for tomato leaf disease classification.

**Figure 2:**
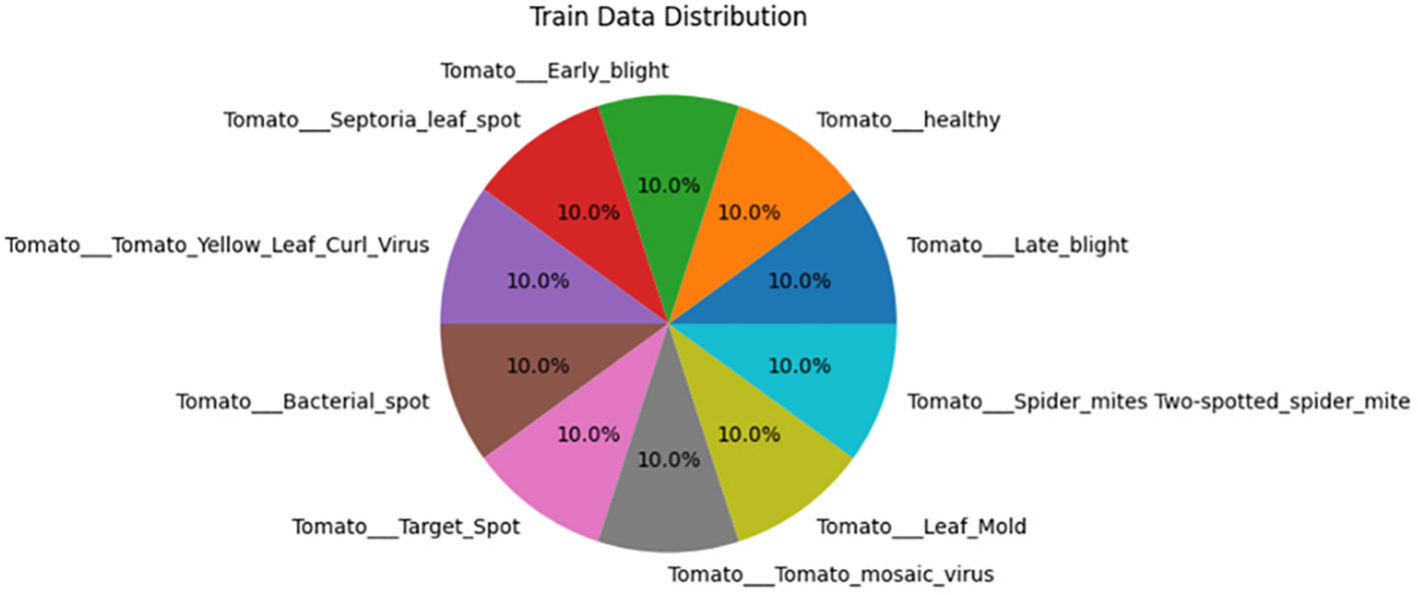
Training data distributions of tomato leaf images.

**Figure 3:**
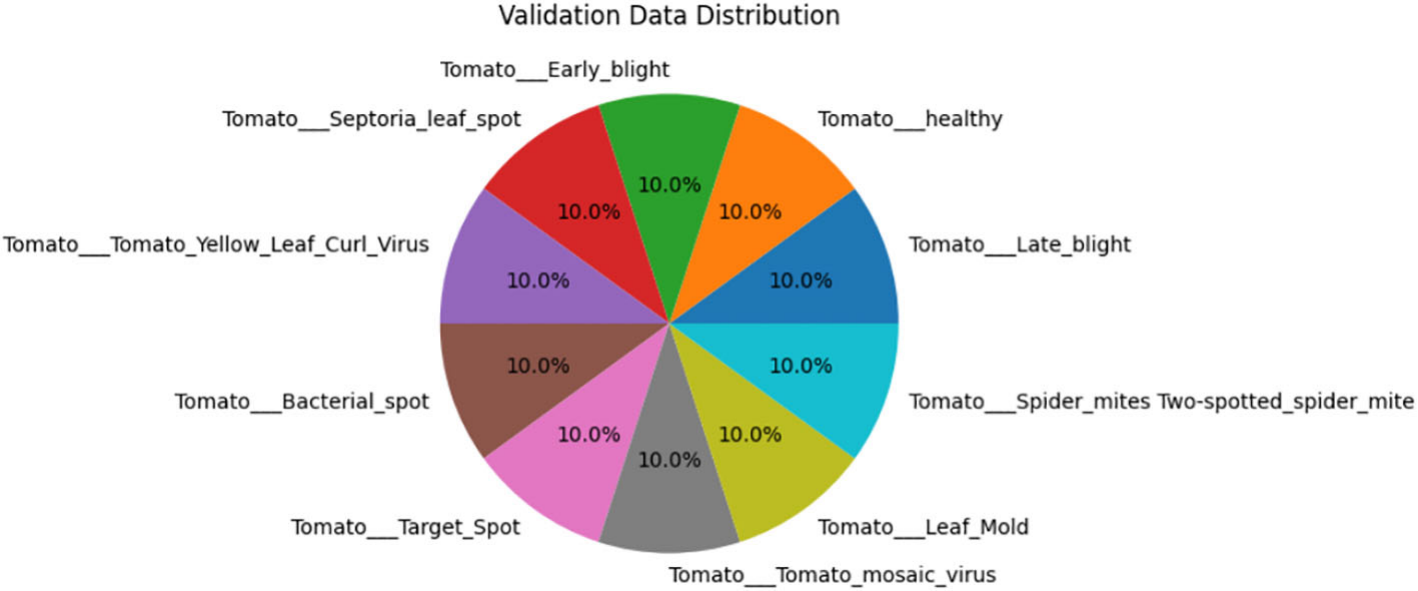
Validation data distributions of tomato leaf images.

**Figure 4:**
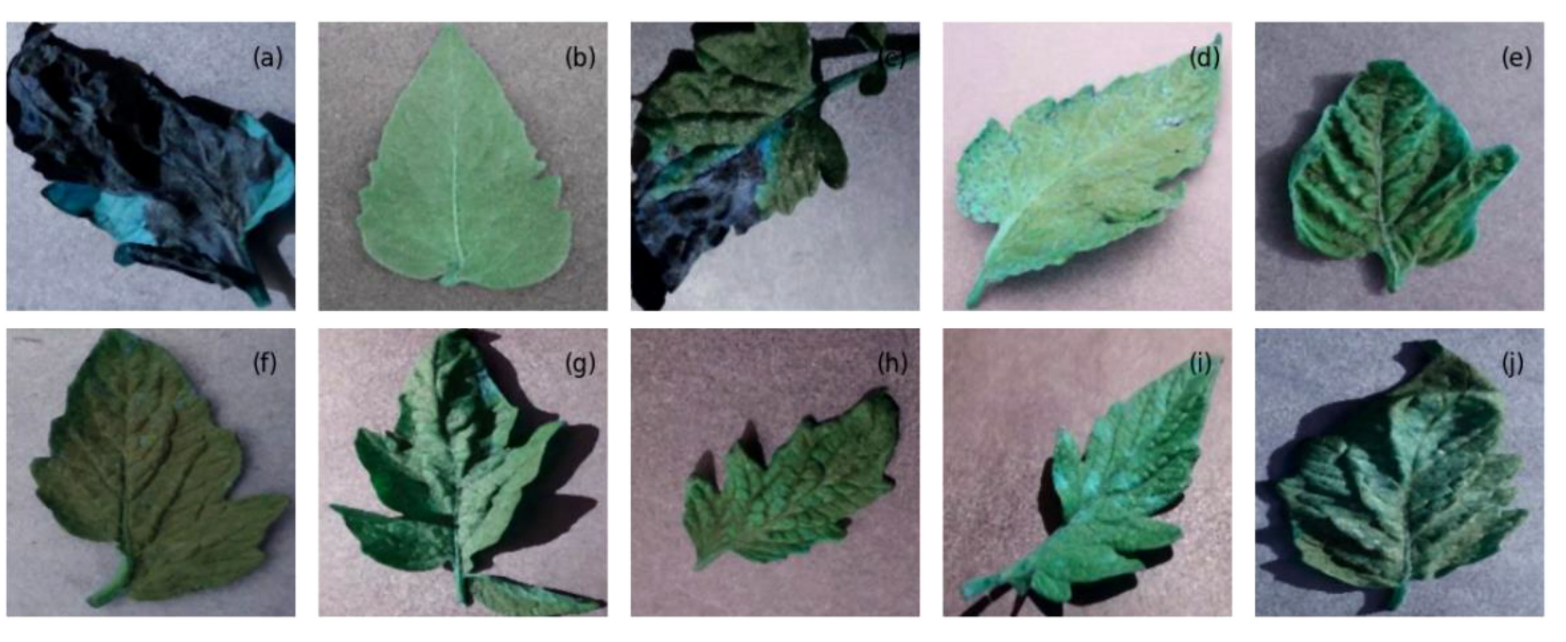
The images from the tomato leaf dataset after preprocessing, representing **(A)** late blight, **(B)** healthy, **(C)** early blight, **(D)** septoria leaf spot, **(E)** yellow leaf curl virus, **(F)** bacterial spot, **(G)** target spot, **(H)** mosaic virus, **(I)** leaf mold, and **(J)** two spotted spider mite.

**Figure 5:**
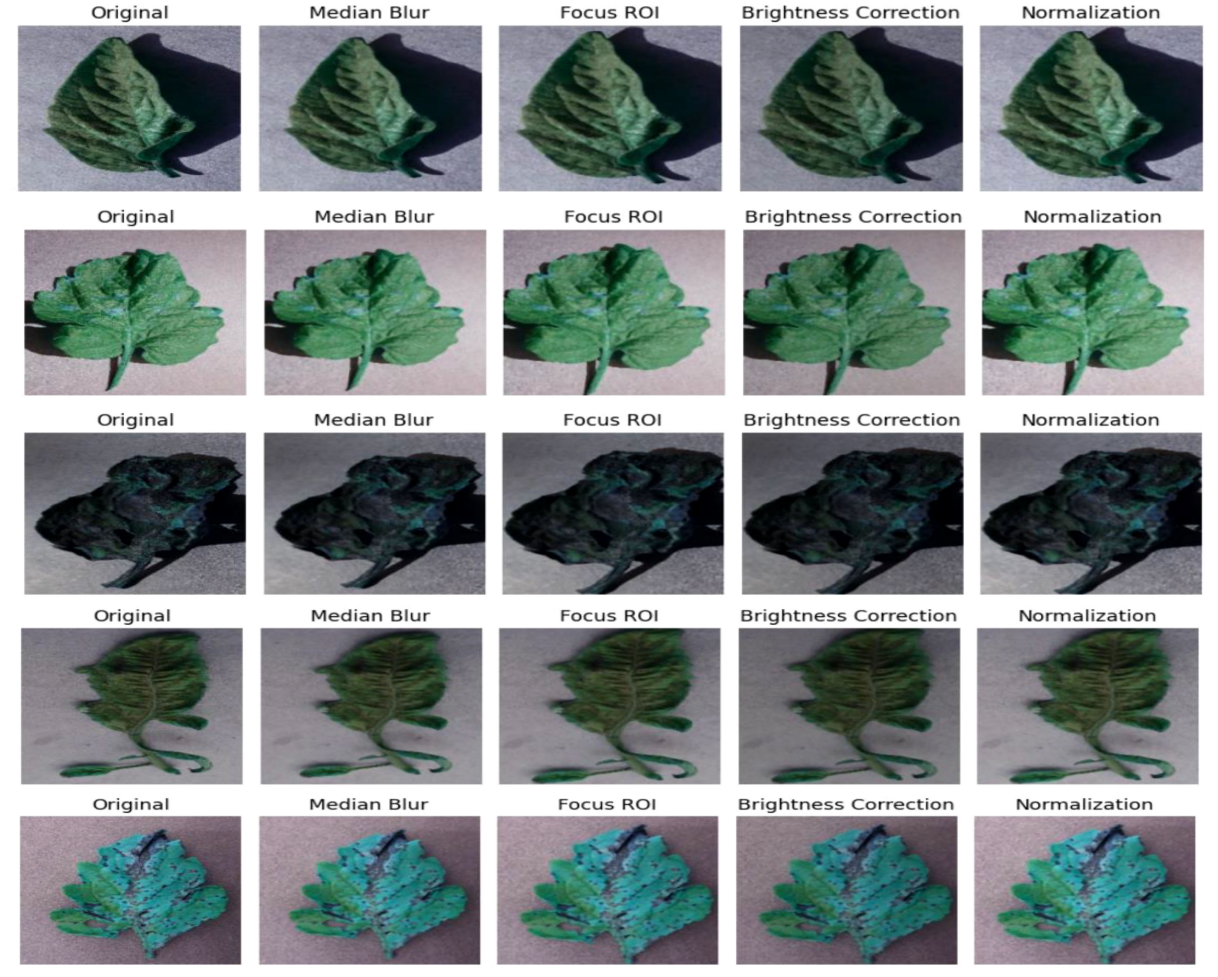
Images of tomato leaves observed at each preprocessing step.

**Figure 6:**
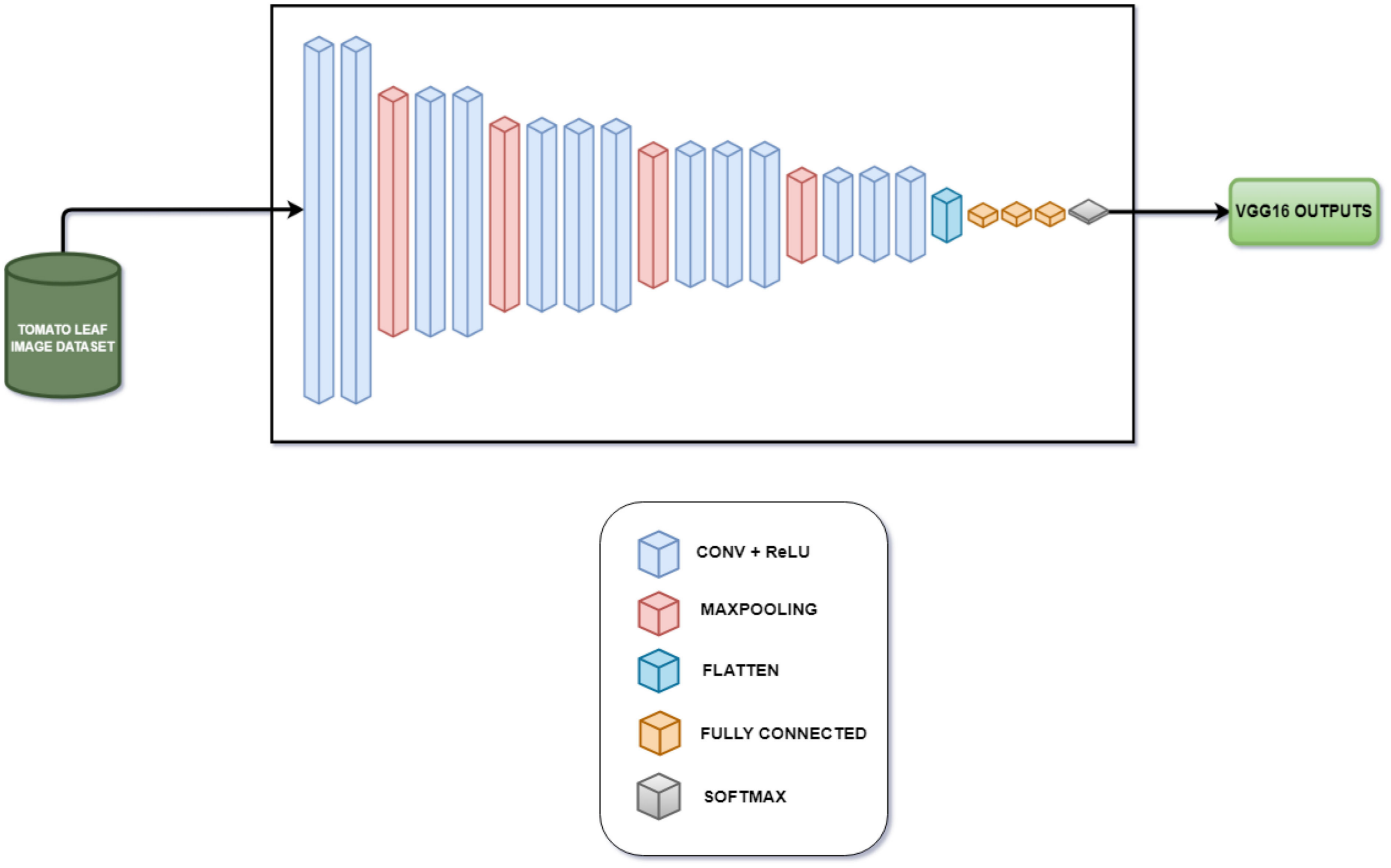
Layer architecture for VGG16 tomato leaf disease classifier.

**Figure 7:**
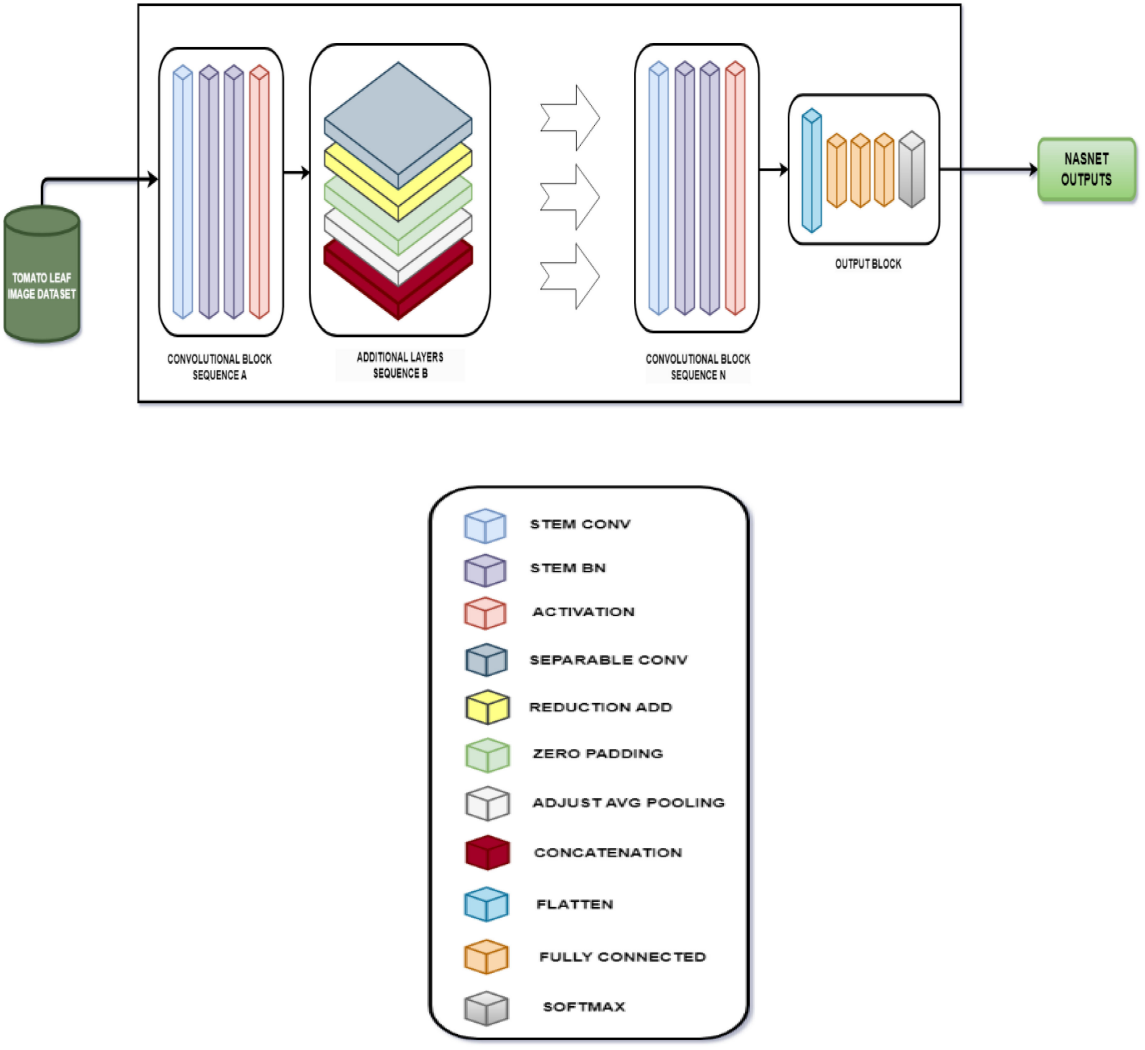
Layer architecture for NASNet mobile tomato leaf disease classifier.

**Figure 8:**
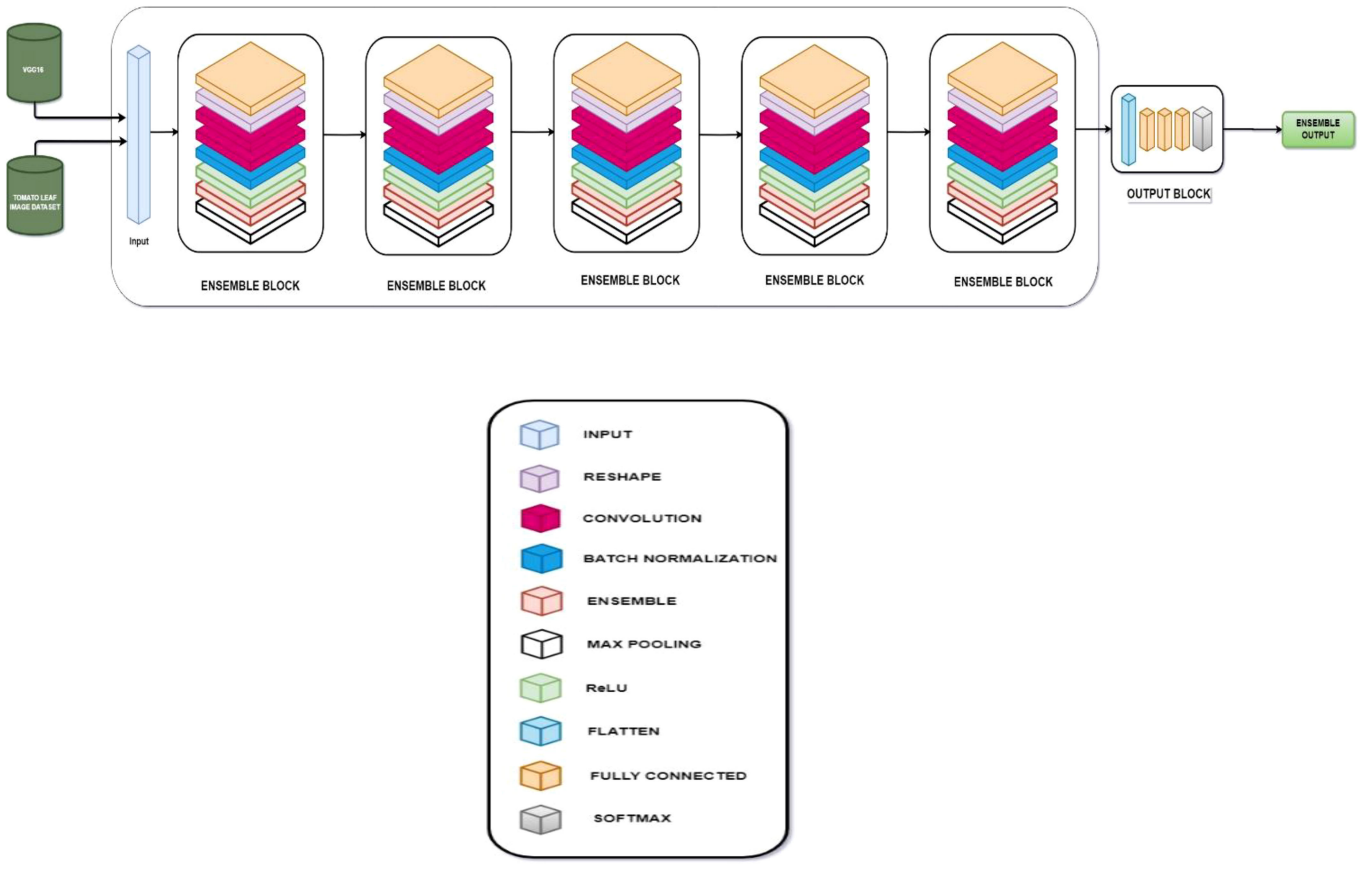
Layer architecture for ensemble model.

The evaluation of the models is robust, using a variety of performance metrics, including the confusion matrix, specificity, accuracy, loss, precision, recall, F1-score, ROC curve, AUC, and misclassification rate. These metrics provide a comprehensive assessment of the model’s effectiveness, making it clear that the research is backed by rigorous analysis and empirical evidence. The overall proposed architecture is shown in **Figure 1**, the training data distributions of the dataset is shown in **Figure 2**, the validated data distributions is shown in **Figure 3**, images of dataset after preprocessing is shown in **Figure 4**, images of tomato leaves observed at each preprocessing step in shown in **Figure 5**, layer architecture for VGG-16 tomato leaf disease classifier is shown in **Figure 6**, layer architecture for NASNet mobile tomato leaf disease classifier is shown in **Figure 7** and layer architecture for ensemble model is shown in **Figure 8**.

#### 4.1.1 Confusion matrices

The confusion matrix is an *n* × *n* matrix where the rows represent the actual classes while the columns represent the predicted class. The data points are stored in the matrix in cells corresponding to the specific actual class and specific predicted class as count values.

**Figure 9:**
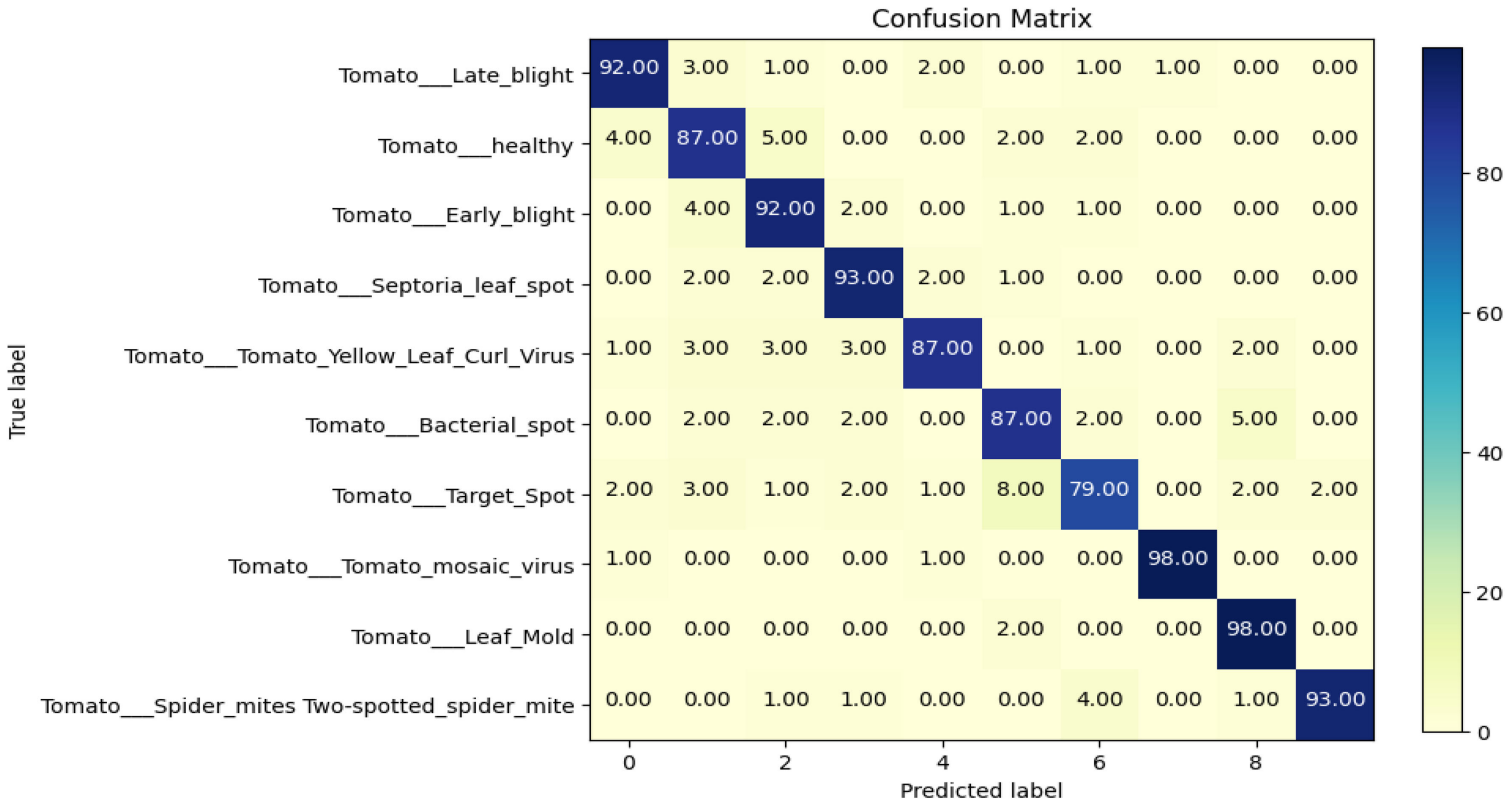
Confusion matrix of the proposed model.

The above confusion matrix consists of the values predicted by the proposed model corresponding to the actual value. The confusion matrix of the proposed model is shown in **Figure 9**, the confusion matrix of the VGG-16 fine-tuned model is shown in **Supplementary Figure 2**, the confusion matrix of the NASNet model is shown in **Supplementary Figure 3**, the precision values of VGG-16, NASNet, and the proposed model is shown in **Supplementary Figure 4**.

#### 4.1.2 Specificity

The specificity is the ratio of true negatives to the actual number of negative instances in a specific class. This is a metric to measure the ability of the classifier for correct identification of negative instance within a specific class.

**Figure 10:**
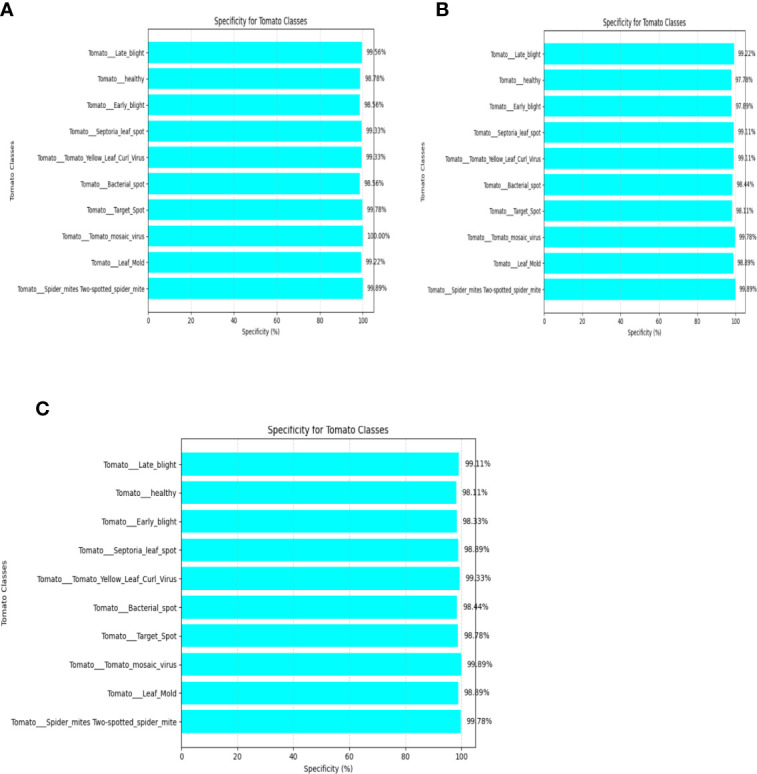
Specificity of **(A)** VGG-16, **(B)** NASNet, **(C)** proposed model.

It is mathematically expressed as shown in Equation (29): Specificity of VGG-16, NASNet, and the proposed model is shown in **Figure 10**.


(29) $$\text{Specificity}=\frac{\sum_{k=1}^{n}TN_{k}}{\sum_{k=1}^{n}\left(TN_{k}+FP_{k}\right)}$$

#### 4.1.3 Accuracy

**Figure 11:**
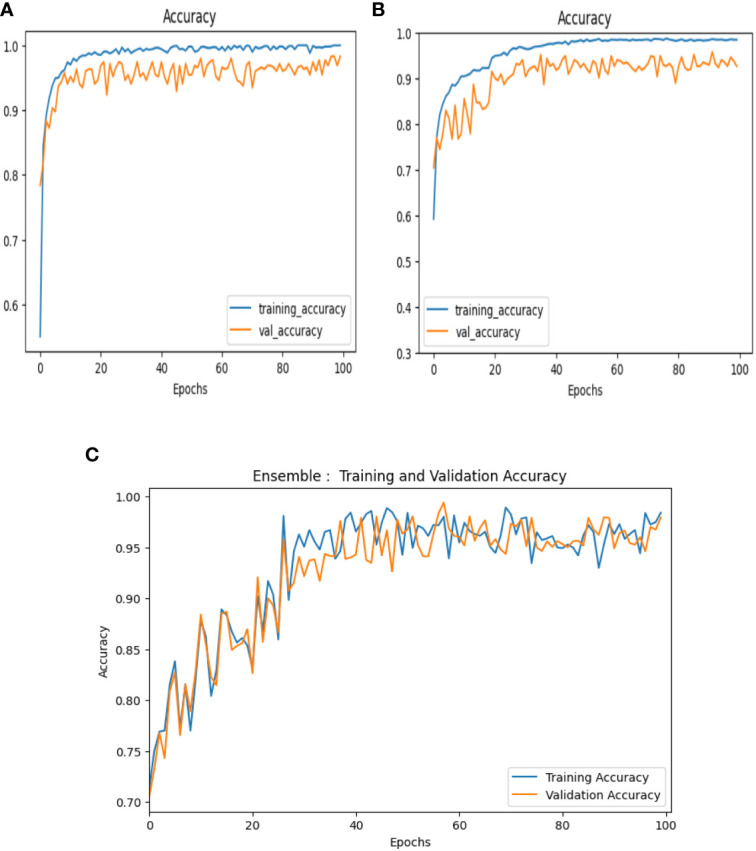
Accuracy curves of **(A)** VGG-16, **(B)** NASNet, **(C)** proposed model.

Accuracy can be defined as the number of correctly classified images to the total number of images in the dataset. This can be expressed mathematically as shown in Equation (30): Accuracy curves of VGG-16, NASNet, and the proposed model is shown in **Figure 11**.


(30) $$\text{Accuracy}=\frac{\sum_{k=1}^{n}\left(TP_{k}+TN_{k}\right)}{\sum_{k=1}^{n}\left(TP_{k}+TN_{k}+FP_{k}+FN_{k}\right)}$$

#### 4.1.4 Loss

**Figure 12:**
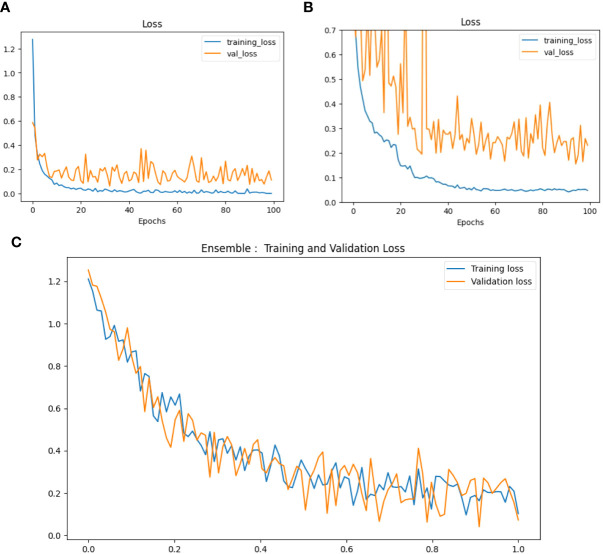
Loss curves of **(A)** VGG-16, **(B)** NASNet, **(C)** proposed model.

Loss is represented as the measure of the model’s performance regarding the ability to minimize the difference between the predicted and actual values. In our case, we have used the categorical cross-entropy loss function. Loss Curves of VGG-16, NASNet and the proposed Model are shown in **Figure 12**.


(31) $$Precision=\frac{\sum_{k=1}^{n}TP_{k}}{\sum_{k=1}^{n}\left(TP_{k}+FP_{k}\right)}$$

#### 4.1.5 Precision

Precision is calculated as the ratio of the true total number of instances that are correctly identified as positive by the classifier to the total number of instances identified as positive by the classifier. This is mathematically expressed as shown in Equation (31):


(32) $$Recall=\frac{\sum_{k=1}^{n}TP_{k}}{\sum_{k=1}^{n}\left(TP_{k}+FN_{k}\right)}$$

#### 4.1.6 Recall

**Figure 13:**
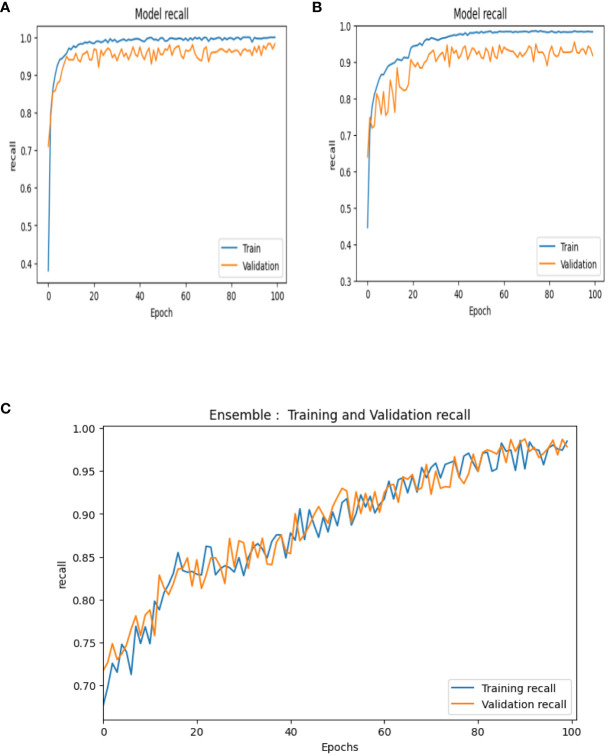
Recall curves of **(A)** VGG-16, **(B)** NASNet, **(C)** proposed model.

Recall or sensitivity is the ratio of the number of true positives to the sum of the number of true-positive and false-negative instances in a specific class. This is a metric to measure the ability of the classifier for correct identification of positive instances within a specific class. The recall curves of VGG-16, NASNet, and the proposed model is shown in **Figure 13**.

It is mathematically expressed as shown in Equation (32):


(33) $$F1\;Score=\frac{2\sum_{k=1}^{n}TP_{k}}{\sum_{k=1}^{n}\left(2TP_{k}+TN_{k}+FP_{k}\right)}$$

#### 4.1.7 F1-score

**Figure 14:**
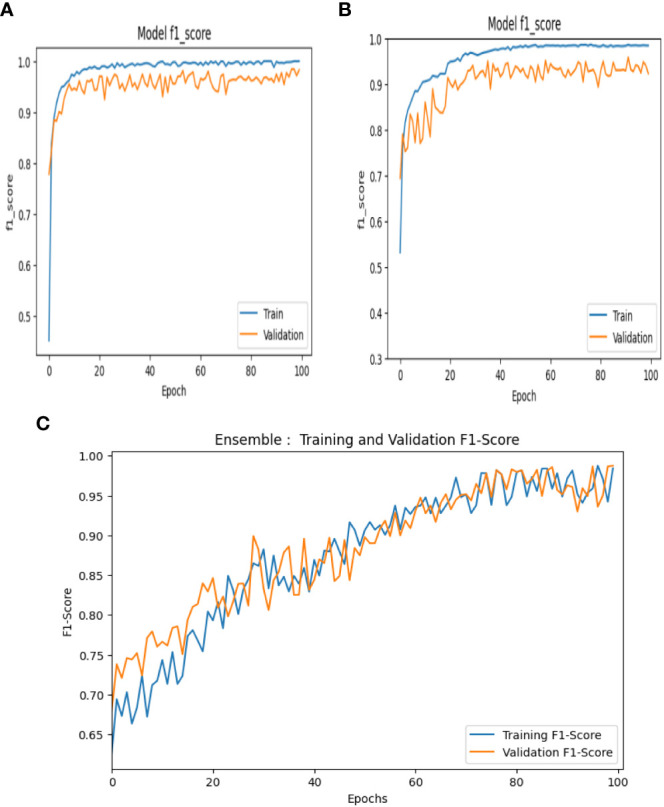
F1 score curves of **(A)** VGG-16, **(B)** NASNet, and **(C)** the proposed model.

The F1-score is utilized for striking a balance between minimizing the false positives and false negatives and is used as a combination of both precision and recall. Thus, it can be mathematically expressed as shown in Equation (33) and the F1 score curves of VGG-16, NASNet, and the proposed model is shown in **Figure 14**.

#### 4.1.8 ROC curve and AUC

**Figure 15:**
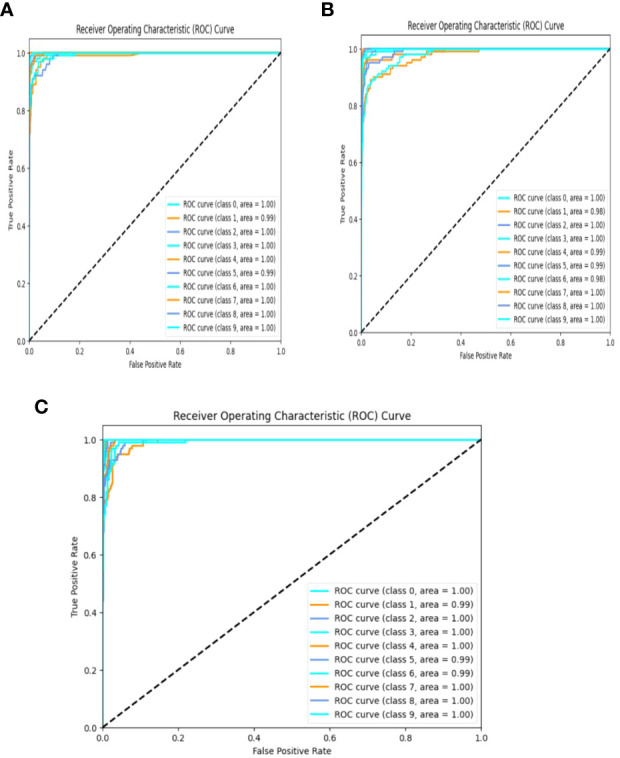
ROC curves of **(A)** VGG-16, **(B)** NASNet, and **(C)** the proposed model.

The receiver operating characteristic (ROC) curve is a graphical representation that consists of the performance of the model in various classification thresholds and is plotted with sensitivity against specificity, thereby visualizing the trade-off between both metrics. AUC helps in quantifying the overall performance of the classifier, which is measured as the area under the ROC curve and the ROC curves of VGG-16, NASNet, and the proposed model is shown in **Figure 15**.

#### 4.1.9 Misclassification rate

**Figure 16:**
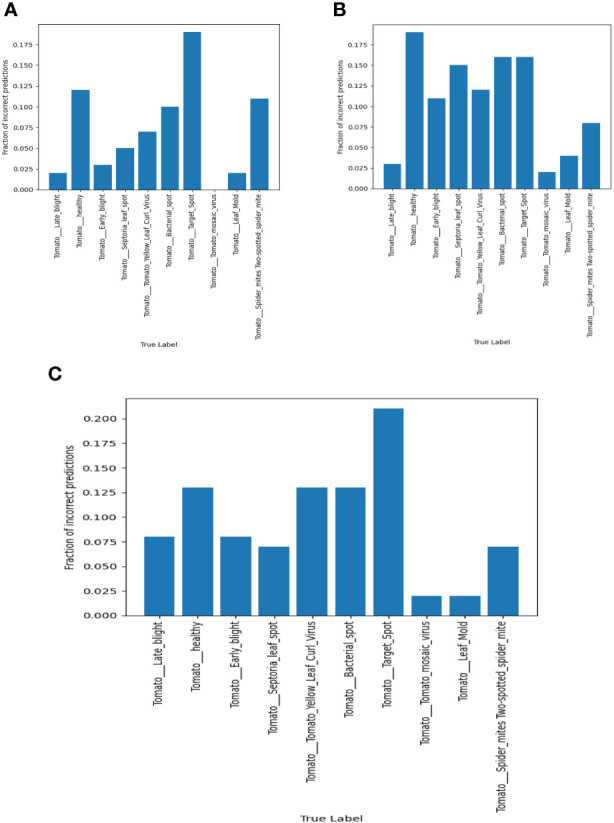
Misclassification rates in **(A)** VGG-16, **(B)** NASNet, **(C)** proposed model.


The error rate can be defined as the number of inputs in a particular, which are classified into a wrong class; this can be expressed mathematically as shown in Equation (34): Misclassification rates in VGG-16, NASNet, and proposed model is shown in **Figure 16**.

(34) $$Error\%=\frac{No\;of\;Misclassified\;Instances\;in\;a\;class}{Total\;Number\;of\;Instances\;in\;a\;class}$$

### 4.2 Performance analysis

The comparison of the three models in the context of the above explained metrics, namely, (a) VGG-16, (b) NASNet, and (c) proposed model, is presented below in graphical representations.

### 4.3 Interpretation

The above computed performance metrics and the respective graphical representations are proof that the proposed deep learning technique, the suitable application of the ensemble model, and the enhanced classifier and optimizer used have shown a tangible increase of the feasibility in the disease prediction for the given series of input images of tomato leaves. It also proves that the preprocessing procedure applied is a fitting one. The performance values observed for accuracy, loss, precision, recall, ROC, and F1-score are 98.7%,<4%, 97.9%, 98.6%, 99.97%, and 98.7% respectively. It is apparent that the results obtained show significant improvement compared with those shown by conventional and present techniques as explained in the literature. The performance scores recorded for the existing models in the literature are tabulated below. The techniques studied do not record all the performance metrics as in the proposed model in this work. One parameter that is considered in all the models, namely, “accuracy”, is exponentially high in the proposed approach. The performance comparison of the proposed model with existing models is shown in **Table 1**.

**Table 1:** Performance comparison.

Models	Performance scores (all in %)							
	Specificity	Accuracy	Recall	Precision	F1-score	Loss	ROC	Misclassification
AlexNet (Wang et al., 2017)	–	91.00	91.00	91.0	91.00	**-**	**-**	**-**
GoogLeNet (Wang et al., 2017)	–	94.8	94	94	94	**-**	**-**	**-**
VGG-16 (Wang et al., 2017)	–	95	95	95	95	**-**	**-**	**-**
VGG-16 (Bracino et al., 2020)	–	90.40	**-**	**-**	**-**	**-**	**-**	**-**
LBP M-SVM (Wang et al., 2017)	90.23	97.20	90.75	93.50	**-**	**-**	**-**	**-**
GPR Quadratic SVM (Ashwinkumar et al., 2022)	–	83.30	**-**	**-**	**-**	**-**	86.00	**-**
OMCNN (Khan et al., 2019)	–	98.7	98.2	**-**	98.5	**-**	–	**-**
**Proposed adaptive ensemble model**	**98.9**	**98.7**	**98.6**	**97.9**	**98.7**	**<4**	**99.97**	**<9**

### 4.4 Testing of hypotheses

In order to provide a statistical analysis on the proposed work, testing of hypothesis was carried out in this work. It consists of three hypotheses including a Null hypothesis given in **Table 2**.

**Table 2:** H0: There is a significant influence between season and tomato leaf diseases.

Reason for tomato leaf disease	Weighted mean using experiments (observed value *O*)	Weighted mean based on computation (expected value *E*)	(*O* − *E*)^2^	Value is ${\text{χ}}^{2}={\text{χ}}^{2}\sum \frac{{\left(O-E\right)}^{2}}{E}$	*p*-value (with 6 dof)
Fungi	7.692	4.649	0.649	4.11	0.65
Fertilizer use	6.329	3.548	0.779		
Bacteria	7.947	4.979	0.612		
Virus	7.309	3.648	0.999		
Viroids	7.519	4.718	0.60		
Geographical Region	6.418	4.269	0.499		

Hypothesis 1: There is a significant influence between season and tomato leaf diseases.

Hypothesis 2: There is no relationship between the occurrence of tomato leaf disease and the environment.

### 4.5 Testing of Hypothesis 1

As the *p*-value in this test is greater than 0.01, the given null hypothesis can be accepted at the 1% significance level. Hence, there is a significant influence between season and tomato leaf diseases.

**Table 3** shows the chi-square test for analyzing the relationship between the deep learning classifier vs. tomato leaf disease detection.

**Table 3:** Analysis of deep learning algorithm’s role in tomato leaf disease detection.

Important metric applied on the algorithm	Chi-square value	*p*-value	Mean availability	
			Up to 80%	Above 80%
Accuracy of classification	1.91	0.41	25	11

### 4.6 Testing of Hypothesis 2

H0: There is no relationship between the selection of the deep learning classifier vs. tomato leaf disease detection for performing accurate detection of the disease.

Since the value of *p* is less than 0.5, this hypothesis, which is shown in **Table 3**, is rejected at the 5% significance level. Therefore, it is concluded that there is a strong and direct relationship between the selection of the deep learning classifier and tomato leaf disease detection from tomato leaf images for performing accurate detection of the disease.

## 5 Conclusion and future work

In this research paper, a new ensemble classifier along with an EMA function with temporal constraints, an EWGO that is integrated with two CNN models, namely, VGG-16 and NASNet, has been proposed for the effective detection of diseases in tomato leaves at an early state. This integration of state-of-the-art deep learning CNN technologies with a gradient optimizer and EMA function with temporal constraints provides meticulous data analysis. The proposed model uses image enhancement techniques, and groundbreaking ensemble models underscore a comprehensive approach to tomato leaf disease classification. The amalgamation of image preprocessing, transfer learning, and the pioneering ensemble model with EWGO exhibits promising outcomes in disease classification and increases detection accuracy compared with the existing systems. The main limitation of this work is the lack of time during training. However, an optimizer is added to this work to solve the training time problem. In the future, the implications of this research shall be extended to areas like crop health, global food security, sustainable agriculture, and environmental preservation, underscoring its value within the realm of plant pathology and agriculture.

## Data availability statement

The original contributions presented in the study are included in the article/**Supplementary Material**. Further inquiries can be directed to the corresponding author.

## Author contributions

PV: Conceptualization, Methodology, Writing – original draft, Writing – review & editing. AS: Data curation, Investigation, Writing – original draft, Writing – review & editing. JP: Methodology, Supervision, Writing – original draft, Writing – review & editing. SV: Software, Visualization, Writing – original draft, Writing – review & editing. SK: Software, Visualization, Writing – original draft, Writing – review & editing. SA: Data curation, Writing – original draft, Writing – review & editing. AA: Software, Visualization, Writing – original draft, Writing – review & editing. AK: Data curation, Writing – original draft, Writing – review & editing.
