# Supplementary material for: Improved tomato leaf disease classification through adaptive ensemble models with exponential moving average fusion and enhanced weighted gradient optimization
Source: Front Plant Sci. 2024 May 17;15:1382416. doi: 10.3389/fpls.2024.1382416 (PMC11140105; doi:10.3389/fpls.2024.1382416)
Supplement: Supplementary file 1 [file DataSheet_1.docx]

**Supplementary Table 1. Tomato Leaf Image Information**

| **Type of tomato leaf disease** | **Training Images** | **Validation Images** | **Test (Epoch)** |
| --- | --- | --- | --- |
| Late Blight | 750 | 250 | 100 |
| Healthy | 750 | 250 | 100 |
| Early Blight | 750 | 250 | 100 |
| Septoria Leaf Spot | 750 | 250 | 100 |
| Yellow Leaf Curl Virus | 750 | 250 | 100 |
| Bacterial Spot | 750 | 250 | 100 |
| Target Spot | 750 | 250 | 100 |
| Mosaic Virus | 750 | 250 | 100 |
| Leaf Mold | 750 | 250 | 100 |
| Two Spotted Spider Mite | 750 | 250 | 100 |

| 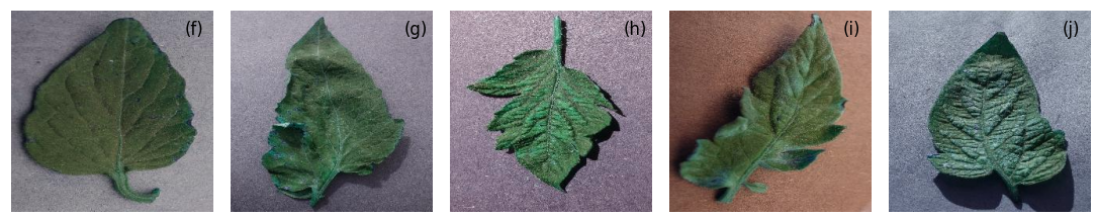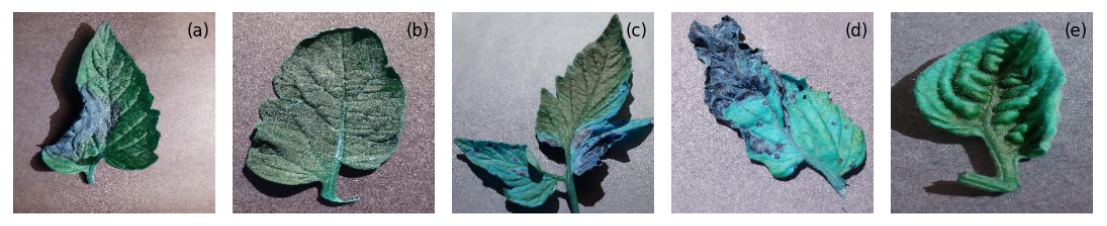 | | |
| --- | --- | --- |
| **Supplementary Figure 1 : Classification of Input Images of Tomato Leaves representing (a) Late Blight, (b) Healthy, (c) Early Blight, (d) Septoria Leaf Spot, (e) Yellow Leaf Curl Virus, (f) Bacterial Spot, (g)Target Spot, (h) Mosaic Virus, (i) Leaf Mold, (j) Two Spotted Spider Mite**  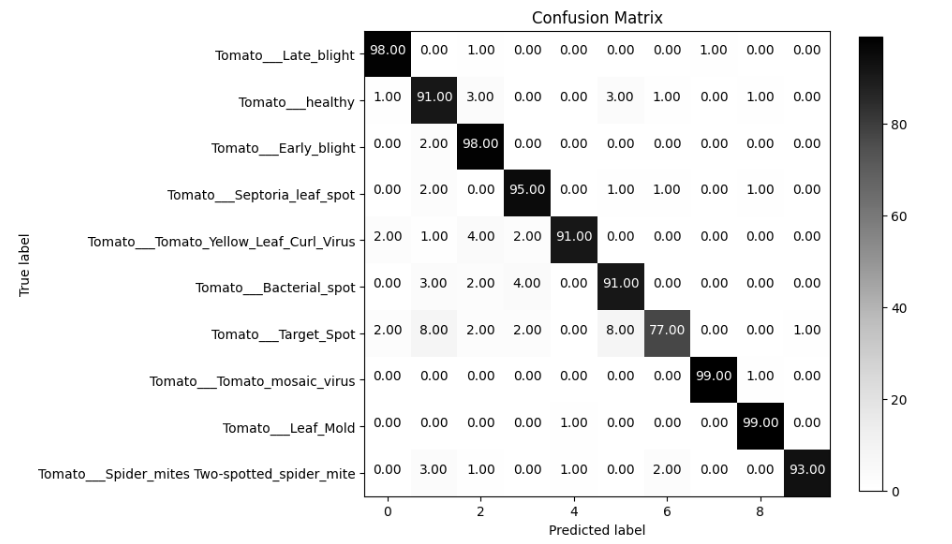  **Supplementary Figure 2. Confusion Matrix of the VGG16 Fine Tuned Model**  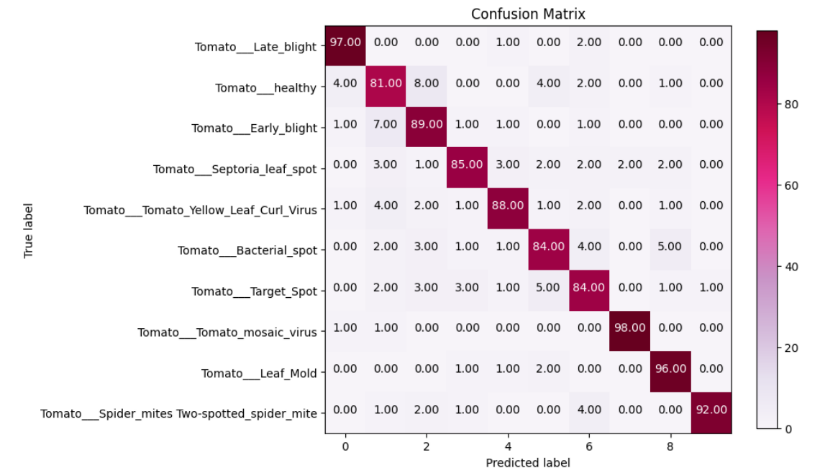  **Supplementary Figure 3. Confusion Matrix of the NASNet model** | | |
| 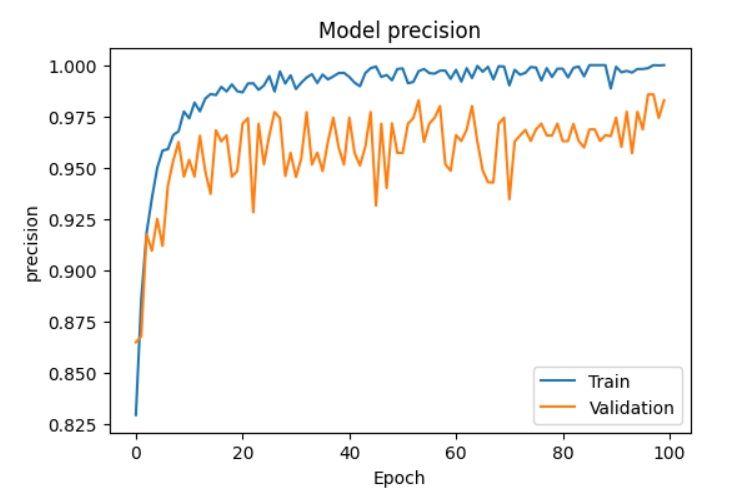  (a) | | 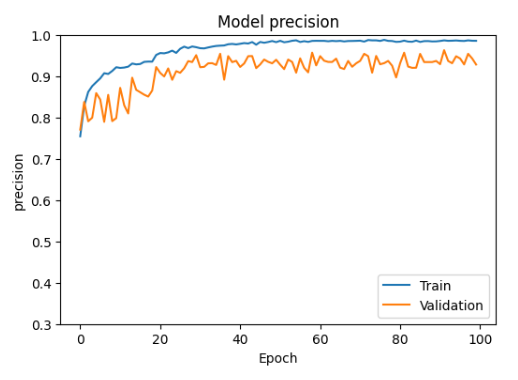 (b) |
| 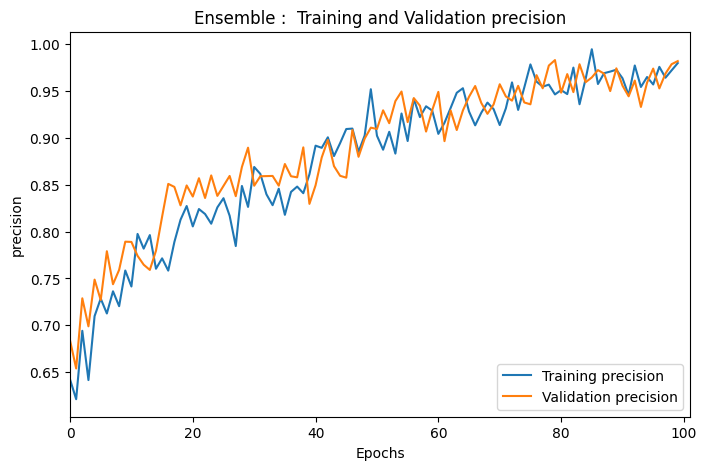  (c) | | |
| **Supplementary Figure 4. Precision Curves of (a) VGG-16, (b) NASNet, (c) Proposed Model** | | |
